# Identification of Alfalfa *SPL* gene family and expression analysis under biotic and abiotic stresses

**DOI:** 10.1038/s41598-022-26911-7

**Published:** 2023-01-03

**Authors:** Yizhen Wang, Qian Ruan, Xiaolin Zhu, Baoqiang Wang, Bochuang Wei, Xiaohong Wei

**Affiliations:** 1 College of Life Science and Technology, Gansu Agricultural University, Lanzhou, 730070 China; 2 Gansu Provincial Key Laboratory of Aridland Crop Science, Gansu Agricultural University, Lanzhou, 730070 China; 3 College of Agronomy, Gansu Agricultural University, Lanzhou, 730070 China

**Keywords:** Molecular biology, Plant sciences

## Abstract

The SQUAMOSA promoter binding-like protein (SPL) is a specific transcription factor that affects plant growth and development. The *SPL* gene family has been explored in various plants, but information about these genes in alfalfa is limited. This study, based on the whole genome data of alfalfa *SPL*, the fundamental physicochemical properties, phylogenetic evolution, gene structure, cis-acting elements, and gene expression of members of the *MsSPL* gene family were analyzed by bioinformatics methods. We identified 82 SPL sequences in the alfalfa, which were annotated into 23 genes, including 7 (30.43%) genes with four alleles, 10 (43.47%) with three, 3 (13.04%) with two, 3 (13.04%) with one allele. These *SPL* genes were divided into six groups, that are constructed from *A. thaliana*, *M. truncatula* and alfalfa. Chromosomal localization of the identified *SPL* genes showed arbitary distribution. The subcellular localization predictions showed that all MsSPL proteins were located in the nucleus. A total of 71 pairs of duplicated genes were identified, and segmental duplication mainly contributed to the expansion of the *MsSPL* gene family. Analysis of the Ka/Ks ratios indicated that paralogs of the *MsSPL* gene family principally underwent purifying selection. Protein–protein interaction analysis of MsSPL proteins were performed to predict their roles in potential regulatory networks. Twelve cis-acting elements including phytohormone and stress elements were detected in the regions of *MsSPL* genes. We further analyzed that the *MsSPLs* had apparent responses to abiotic stresses such as drought and salt and the biotic stress of methyl jasmonate. These results provide comprehensive information on the *MsSPL* gene family in alfalfa and lay a solid foundation for elucidating the biological functions of *MsSPLs*. This study also provides valuable on the regulation mechanism and function of *MsSPLs* in response to biotic and abiotic stresses.

## Introduction

The SBP (SQUAMOSA PROMOTER BINDING PROTEIN), also known as SPL (SQUAMOSA PROMOTER BINDING PROTEIN-LIKE), is a plant-specific transcription factor. The *SBP* gene was first discovered in snapdragon, which can precisely identify the flower development gene SQUAMOSA promoter and regulate gene expression^1^. Members of the *SPL* gene family all have a highly conserved DNA binding domain (SBP) containing about 75-78 amino acid residues, consisting of two typical C3H (C–C–CH), C2HC (C–C–H–C) zinc finger structures, and a nuclear localization signal (NLS), which binds to the GTAC core sequence and functions^2^. SPL not only functions as a transcription factor but also acts as a target gene of *miRNA156/miRNA157* to form a complex network regulation system that affects plant growth and development. Studies have shown that *miR156/miR157* also play an important role in abiotic stresses^3^.

With the development of bioinformatics, members of the *SPL* gene family have been found in increasing number of species for bioinformatics analysis and functional identification, including *Arabidopsis thaliana*^4^, Wheat^5^, Rice^6^, Sugar cane^7^, Populus euphratica^8^, Pecan^9^, Cotton^10^, Castanea mollissima^11^, Betula platyphylla suk^12^ et al. SPL transcription factors play an important role in regulating plant development. It has been shown that *SPL3*, *SPL4*, and *SPL5* in Arabidopsis respond to photoperiod and promote the development of floral meristematic tissues^13^. Most of the *SPLs* found in wheat can regulate the development of inflorescence, spike, ear, and grain, and regulate onset and anthesis^5,14–16^. Alterations in *SPL* gene expression control rice panicle structure, panicle development, and grain size, affect leaf and stem development, regulate plant height and tiller number^6,17–21^. In addition, *SPL* genes play a vital part in coping with biotic and abiotic stresses. Down-regulation of the expression of three target genes *SPL3*, *-14,* and *-17* of *OsmiR156k* and the three target genes *OsSPL14*, *-11,* and *-4* of *OsmiR535* in rice transgenic lines can affect the osmotic regulation under low-temperature stress and reduce the tolerance of rice to cold stress; *SPL10* knockout mutants in rice exhibit enhanced salt tolerance^22–24^. In cabbage, the expression levels of *BoSPL1*, *-9a*, *-9b*, *-10b*, *-11b* in cold-tolerant cabbage and *BoSPL9b*, *-15a*, and *16a* in cold-sensitive cabbage were induced to be up-regulated after low-temperature stress, suggesting that these genes may play a role in the cold tolerance of cabbage^25^. Overexpression of *miR156a* decreased the salt tolerance in apple, whereas overexpression of *MdSPL13* enhanced its salt tolerance^26^. Alfalfa *miR156* targeted *SPL13* gene expression decreased, showed lower water loss, increased stomatal conductance, and improved drought tolerance^27^.

Alfalfa (*Medicago sativa* L.) is a perennial herb belonging to the fabaceae family. It is known as the "king of forage grasses" because of its high protein and vitamin content. Alfalfa suffers from various severe abiotic stresses during its growth, such as salinity, drought, extreme temperature, heavy metals et al. Therefore, continuing research on alfalfa adaptation to stress and the detection of stress resistance-related genes is crucial for promoting the breeding and crop improvement. In recent years, the rapid development of high-throughput sequencing technology and bioinformatics has made it possible to identify and analyze the alfalfa *SPL* gene family at the genome-wide level. In this study, 23 genes were screened from the alfalfa *SPL* gene family and the evolutionary analysis, protein structure, and expression profile of the family members were systematically analyzed. Quantitative Real-Time PCR was used to analyze the differences between drought, salt, and methyl jasmonate stress. The spatio-temporal expression of *SPL* genes in alfalfa leaves at treatment time points is expected to lay a foundation for in-depth studies on the functions of *MsSPLs*.

## Results

### Identification of *MsSPL* gene family in alfalfa

**Figure 1:**
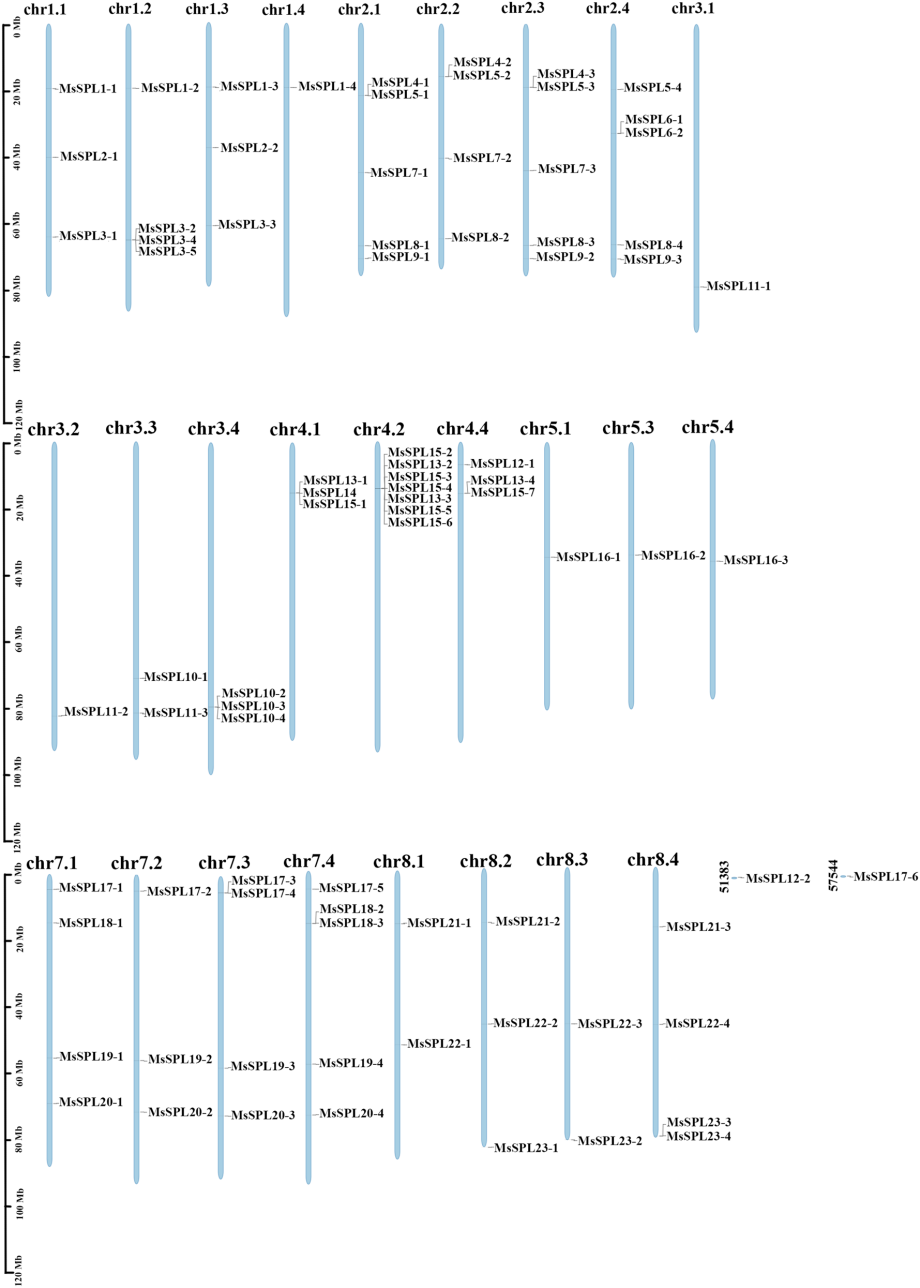
Chromosomal location of the *MsSPLs*. Distribution of the *SPL* gene in the alfalfa genome. The *MsSPLs* are located on chromosomes 1.1-1.4, 2.1-2.4, 3.1-3.4, 4.1-4.2, 4.4, 5.1, 5.3-5.4, 7.1-7.4, and 8.1-8.4. The number of the chromosome is shown at the top of the chromosome. The scale (Mb) represents the length of the chromosome.

Twenty-three *MsSPL* genes were identified from the alfalfa allele haploid database, and these genes were named *MsSPL1-MsSPL23* according to the location of chromosomes (Fig. 1 and Table 1). The allele of each *MsSPL* was named with the gene names of "-1" to "-4", including 7 genes with 4 alleles (30.43%), 10 genes with 3 alleles (43.47%), 3 genes with 2 alleles (13.04%), 3 genes with 1 allele (13.04%), *MsSPL15* have two tandem duplicators. In addition, some genes also contain paralogs from different homologous chromosomes, and these were regarded as different copies of one number of the *SPL* gene family.

**Table 1:** Identification of the alleles and duplicates of *MsSPL* genes in alfalfa.

Gene name	Allele-1	Allele-2	Allele-3	Allele-4	Tandem duplicate	Paralogous
*MsSPL1*	*MsSPL1-1*	*MsSPL1-2*	*MsSPL1-3*	*MsSPL1-4*	–	–
*MsSPL2*	*MsSPL2-1*	–	*MsSPL2-2*	–	–	–
*MsSPL3*	*MsSPL3-1*	*MsSPL3-2*	*MsSPL3-3*	–	–	*MsSPL3-4*
						*MsSPL3-5*
*MsSPL4*	*MsSPL4-1*	*MsSPL4-2*	*MsSPL4-3*	–	–	–
*MsSPL5*	*MsSPL5-1*	*MsSPL5-2*	*MsSPL5-3*	*MsSPL5-4*	–	–
*MsSPL6*	*MsSPL6-1*	–	–	–	–	*MsSPL6-2*
*MsSPL7*	*MsSPL7-1*	*MsSPL7-2*	*MsSPL7-3*	–	–	–
*MsSPL8*	*MsSPL8-1*	*MsSPL8-2*	*MsSPL8-3*	*MsSPL8-4*	–	–
*MsSPL9*	*MsSPL9-1*	–	*MsSPL9-2*	*MsSPL9-3*	–	–
*MsSPL10*	–	–	*MsSPL10-1*	*MsSPL10-2*	–	*MsSPL10-3*
						*MsSPL10-4*
*MsSPL11*	*MsSPL11-1*	*MsSPL11-2*	*MsSPL11-3*	–	–	–
*MsSPL12*	*MsSPL12-1*	–	–	–	–	*MsSPL12-2*
*MsSPL13*	*MsSPL13-1*	*MsSPL13-2*	–	*MsSPL13-4*	–	*MsSPL13-3*
*MsSPL14*	*MsSPL14*	–	–	–	–	–
*MsSPL15*	*MsSPL15-1*	*MsSPL15-2*	–	*MsSPL15-7*	*MsSPL15-3*	*MsSPL15-4*
					*MsSPL15-5*	*MsSPL15-6*
*MsSPL16*	*MsSPL16-1*	–	*MsSPL16-2*	*MsSPL16-3*	–	–
*MsSPL17*	*MsSPL17-1*	*MsSPL17-2*	*MsSPL17-3*	*MsSPL17-5*		*MsSPL17-4*
						*MsSPL17-6*
*MsSPL18*	*MsSPL18-1*	–	–	*MsSPL18-2*	–	*MsSPL18-3*
*MsSPL19*	*MsSPL19-1*	*MsSPL19-2*	*MsSPL19-3*	*MsSPL19-4*	–	–
*MsSPL20*	*MsSPL20-1*	*MsSPL20-2*	*MsSPL20-3*	*MsSPL20-4*	–	–
*MsSPL21*	*MsSPL21-1*	*MsSPL21-2*	–	*MsSPL21-3*	–	–
*MsSPL22*	*MsSPL22-1*	*MsSPL22-2*	*MsSPL22-3*	*MsSPL22-4*	–	–
*MsSPL23*	–	*MsSPL23-1*	*MsSPL23-2*	*MsSPL23-3*	–	*MsSPL23-4*

The basic physicochemical properties of 23 genes including alleles, tandem duplicates and paralogous were shown in supplementary Table S1. The predicted physicochemical properties of amino acid sequences indicated that the 23 *SPL* genes encode proteins containing 72 (*MsSPL5*) to 1026 (*MsSPL7-2*) amino acids with molecular weights ranging from 8447.27 (*MsSPL5-1,-2,-3*) to 113,299.24 Da (*MsSPL1-1*). The overall mean of the hydrophilic (GRAVY) scores of all SPL proteins was negative, indicating that they are all hydrophilic proteins. The predicted that isoelectric points ranged from 5.33 (*MsSPL9-3*) to 10.6 (*MsSPL5*). The subcellular localization predictions showed that all MsSPL proteins were located in the nucleus. The secondary structure prediction of alfalfa SPL proteins showed that random coil is the main component of the secondary structure of MsSPL protein (Supplementary Table S2), accounting for 71.01-45.83%, followed by extended chain and α-helix, accounting for 37.20-11.58% and 33.33-7.71%, respectively. The tertiary structure of MsSPL protein showed that the MsSPL protein family was dominated by random coils (Supplementary Table S2), which was consistent with the prediction of the secondary structure. The modeling and prediction of the tertiary structure of MsSPL proteins provide a theoretical reference for the subsequent research of MsSPL proteins family.

### Chromosome mapping of *SPL* gene in alfalfa

Physical location of 23 genes (82 SPL sequences) were unevenly distributed on 26 chromosomes (2n = 4x = 32) of alfalfa (Fig. 1). The most numerous of them were on chromosome chr 4.2 with 7 SPL sequences, chromosomes chr 1.4, chr 3.1, chr 3.2, chr 5.1, chr 5.3, and chr 5.4 all had only one SPL sequence distribution, and the rest of the chromosomes had a range of two to five SPL sequences.

### Phylogeny of alfalfa SPL protein family

**Figure 2:**
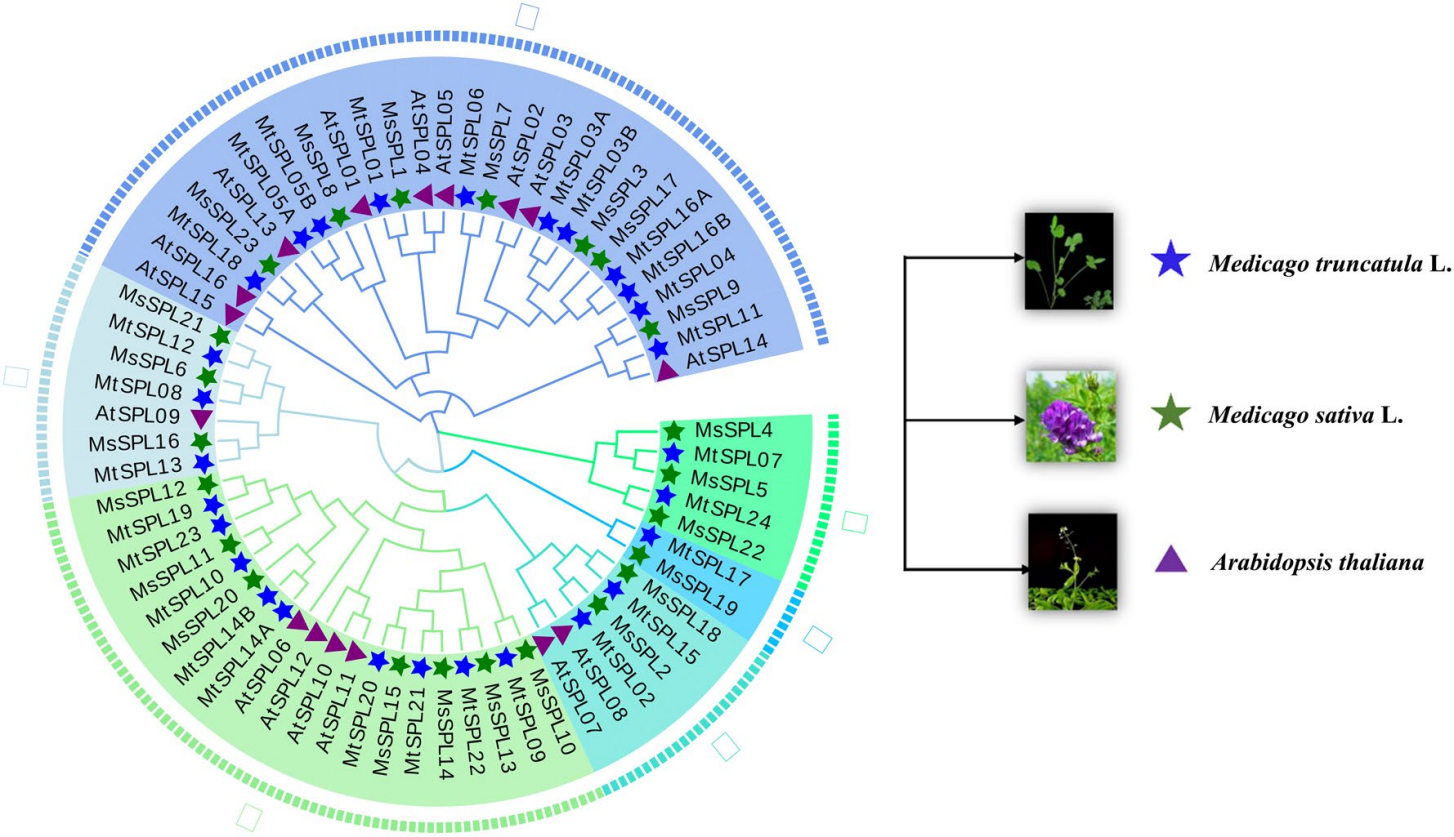
Phylogenetic analysis of *M. sativa*, *M. truncatula* and *A. thaliana*. Proteins with SBP domains from *M. sativa* (Ms), *A. thaliana* (At) and *M. truncatula* (Mt) were searched and named SPL. Different colors represent different clades, green stars mark *SPL* gene family members in *M. sativa*, blue stars mark *SPL* gene family members in *M. truncatula*, and purple triangles mark *SPL* gene family members in *A. thaliana*.

To analyze the evolutionary relationships of the alfalfa SPL protein family, 16 *A. thaliana* SPL proteins from *A. thaliana* and 28 SPL proteins from *M. truncatula* were selected to construct a phylogenetic tree together with 23 alfalfa SPL proteins identified in this study (Supplementary Table S3). Results showed that these SPL proteins were classified into six clades based on phylogenetic relationship (Fig. 2). The remaining 6 subgroups all have MsSPL members, of which the I subgroup contains 27 members, the largest number; the II, III, IV, V, VI subgroups contain 7, 20, 6, 2, 5 MsSPL members. There are a total of 19 direct homologous pairs in the evolutionary tree, including MsSPL9 and MtSPL04, MsSPL7 and MtSPL06, MsSPL1 and MtSPL01, MsSPL23 and MtSPL18, MsSPL21 and MtSPL12, MsSPL6 and MtSPL08 et al. MtSPL16A and MtSPL16B, AtSPL02 and AtSPL03, AtSPL04 and AtSPL05 et al. for a total of 9 parologous homologous pairs. In general, the MsSPLs displayed closer phylogenetic relationships with those of *M. sativa* and *M. truncatula* compared to that of *A. thaliana*.

### *MsSPL* gene structure analysis, conserved motif, and binding domain analysis

**Figure 3:**
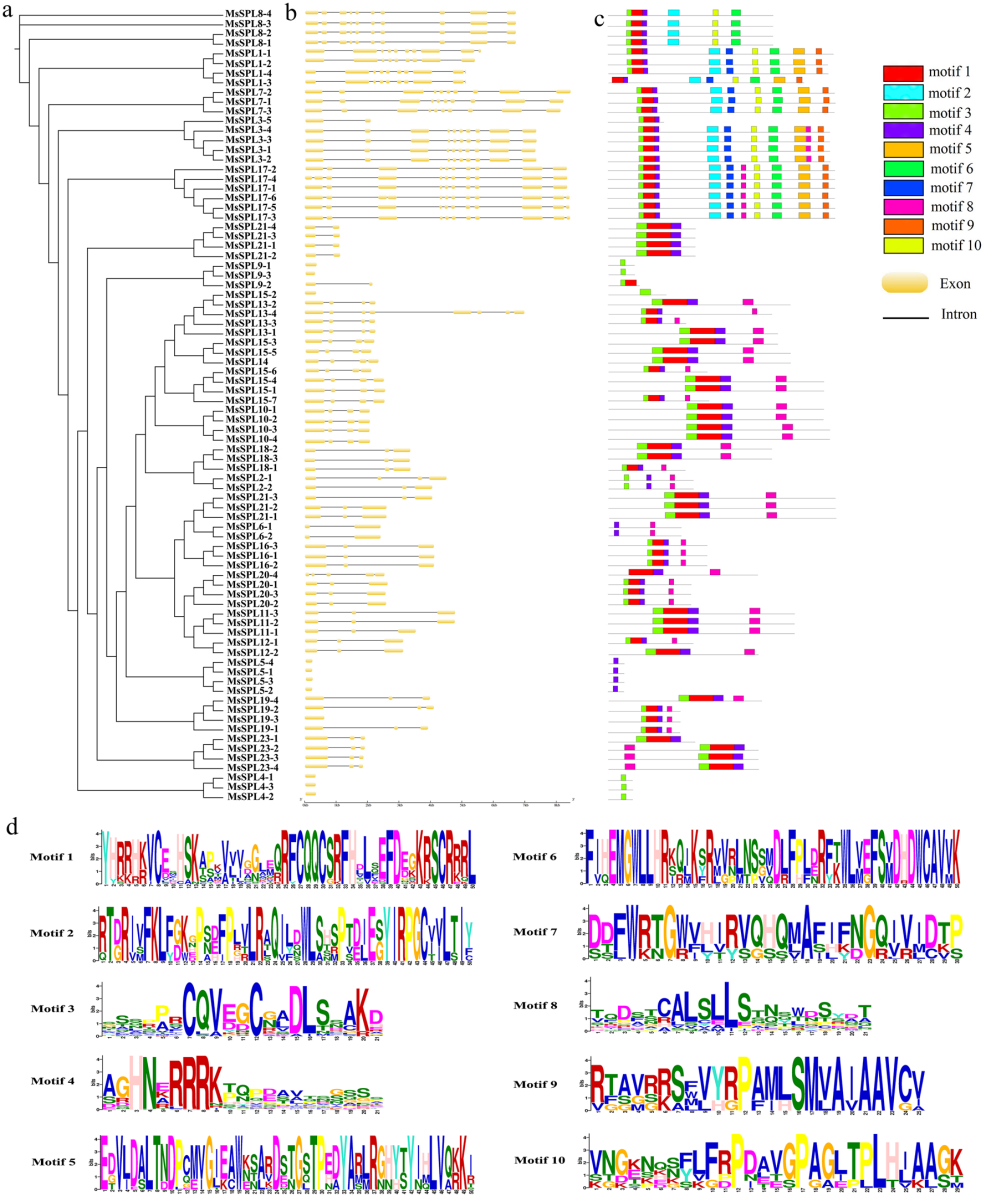
Gene structure and conserved motif analysis. (**a**) Alfalfa SPL evolutionary tree; (**b**) exon-intron structure of the *SPL* genes in alfalfa, the yellow box represents the exon, and the black line connecting the two exons represents the intron; (**c**) the conserved motif in alfalfa, each motif is shown as a box in one of 10 different colors; (**d**) ten conserved motif structures.

Further gene structure analysis was performed on alfalfa *SPL* genes. In the *MsSPL* gene family, the number of introns ranges from 0 to 4 (Fig. 3b), while, 10 out of 82 alleles and paralogs contain no intron, 9 contain one intron, 25 contain two introns and 38 contain more than three introns. The structure of alleles in the same subfamily was similar, but the structure of alleles in different subfamilies was different, which indicated the diversity of gene evolution trend and the diversity of gene structure.

The similarity and diversity of motifs in different MsSPL proteins were explored, MEME software was used to predict and Tbtools to predict the structural protein domains. The results showed that MsSPL2-1 and MsSPL10-1 had two SBP structural domains, the rest had one SBP structural domain (Supplementary Fig. S1). Ten relatively conserved motifs (named motif 1-10) were identified (Fig. 3d), and the conserved motifs of members of the same subfamily were similar, the subfamily with the largest number of exons also contained the most motifs (Fig. 3c). 88% (72) genes of the 82 alleles and paralogs contained multiple motif structures, and 12% (10) genes contained only one motif structure, among which motif 1, motif 3, motif 4, and motif 8 were the most conserved and shared by most MsSPL proteins. In addition to common motifs, each group of motifs also has certain specificity, and there are similar conserved motifs among subfamilies, such as motif 2, motif 6, and motif 10, which are only found in I subgroup. The presence of subfamily-specific conserved motifs in the subfamily may play an critical role in the functional specificity of the subfamilies.

### Analysis of cis-acting elements

**Figure 4:**
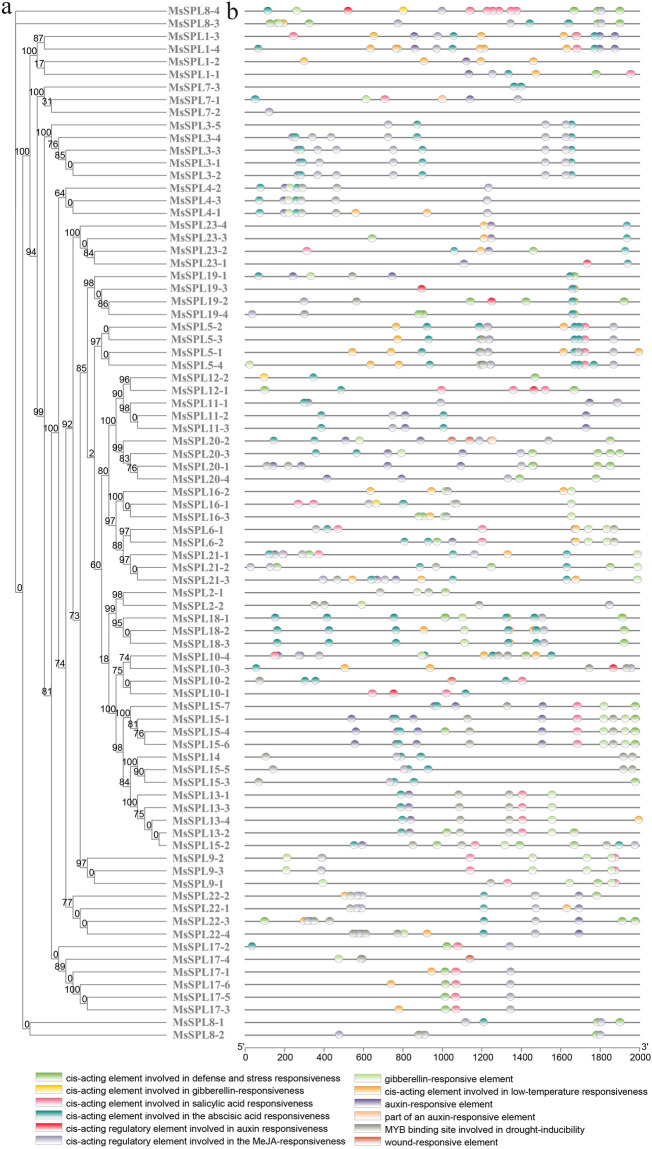
*MsSPL* promoter cis-acting element. (**a**) Alfalfa SPL evolutionary tree; (**b**) distribution of cis-acting elements in promoters of alfalfa *SPL* gene family. Different colored squares show different promoter cis-acting elements.

To further predict the potential functions of the alfalfa *SPL* gene family, the promoter regions of the alfalfa *SPL* gene family were analyzed and found that it contained multiple types of cis-acting elements (Fig. 4), among which the stress response elements included drought, low temperature, wound, and defense and stress element; hormone response elements included growth gibberellin, abscisic acid, salicylic acid, methyl jasmonate response element. As seen in Fig. 4 that the ditribution of cis-element between *MsSPL* alleses promoters is different, indicating that *MsSPL* genes may participate in plant abiotic stress responses through multiple cis-acting elements. Among them, *MsSPL8-4* containd the most cis-acting elements (15), followed by *MsSPL10-2* and *MsSPL1-4*, which contained 14 elements. *MsSPL7-2* had one and only one cis-acting element that responds to methyl jasmonate, and the rest of the genes contained at least one plant hormone response element, including 48 (59%), 35 (43%), 37 (45%), 43 (52%), 66 (80%) *MsSPLs* with one or more methyl jasmonate response elements, salicylic acid response elements, gibberellin responsive elements, auxin-responsive elements, and abscisic acid-responsive elements. In addition, abiotic stress-related genes were enriched in promoter regions, with 37 (45%), 31 (38%) and 39 (48%) *MsSPLs* contained defense and stress response elements, low temperature response elements and drought response elements; *MsSPL10-2*, *MsSPL20-2*, *MsSPL17-4* contained wound-response elements.

### Analysis of SPL protein interaction in alfalfa

**Figure 5:**
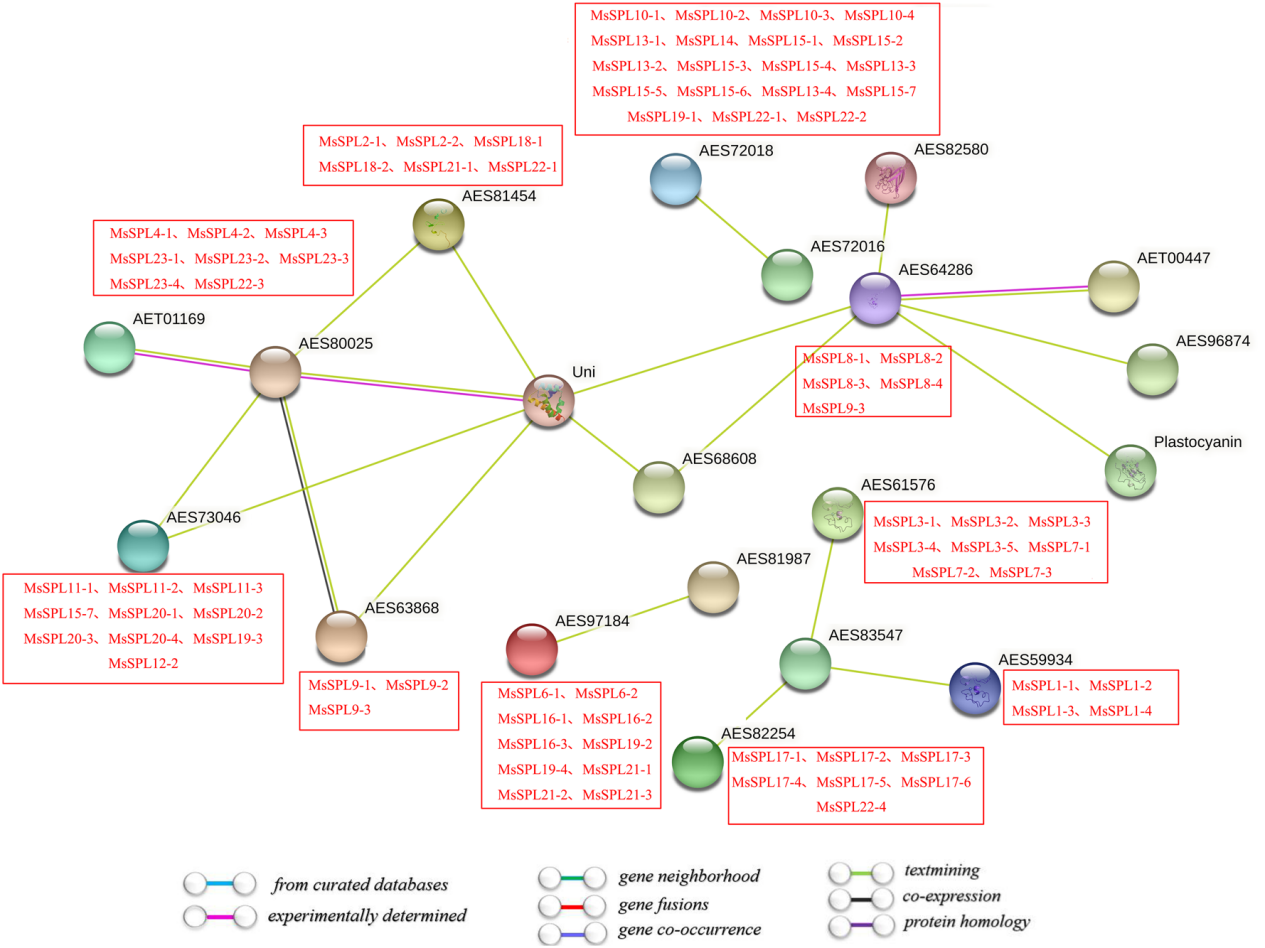
MsSPL proteins interaction network diagram. The color scale represents relative signal intensity fractions.

Using protein network interactions to connect unknown functional proteins into protein interaction networks will help further understand the biological functions of proteins. Therefore, in this study, *M. truncatula* was used as a background to predict the potential interacting proteins associated with the protein function of MsSPLs. The expected number of edges in the interaction network graph was 11, the average local clustering coefficient: was 0.832, the protein-protein interaction enrichment P-value was 0.00948, which was considered reasonable for the results. A total of 10 functional molecules directly related to MsSPL proteins and 10 potential interacting proteins were identified (Table 2), and all 10 interacting proteins were SQUAMOSA promoter-binding proteins (Fig. 5). It has interactions with superoxide dismutase, floral meristem control frond (LFY) protein, Ubiquitin-conjugating enzyme, MADS-box transcription factor growth regulator, DNA/RNA binding protein kin17, eukaryotic translation initiation factor 2c, plastocyanin et al. have an interaction relationship. It is speculated that the MsSPL proteins may be synergistically involved in the process of disease resistance and stress resistance in plants with these interacting proteins.

**Table 2:** MsSPL functional protein-protein interactions.

Interaction gene	Gene description	Aminoacid number	Interaction coefficient
AES82580	supe roxide dismutase [Cu-Zn] protein	152aa	0.870
Uni	Floral meristem identity control protein LEAFY (LFY) protein	386aa	0.862
AES80025	MADS-box transcription factor	213aa	0.860
AES81987	growth-regulating factor	384aa	0.842
AET00447	DNA/RNA-binding protein Kin17, motif protein	398aa	0.834
AES68608	FAR-RED ELONGATED HYPOCOTYL	844aa	0.819
AES96874	eukaryotic translation initiation factor 2c	1016aa	0.798
Plastocyanin	Participates in electron transfer between P700 and the cytochrome b6-f complex in photosystem I	139aa	0.776
AES72016	F-box only-like protein	465aa	0.768
AES83547	Ubiquitin carrier protein	161aa	0.763

### Analysis of the duplication events of the alfalfa *MsSPL* gene

Gene duplication is one of the important mechanisms for plants to acquire and create new genes, and it is also the main driving force in the evolution of genomes. Gene duplication usually has two duplication modes: segmental duplication and tandem duplication. The alfalfa *MsSPL* gene duplication event was studied, tandem duplication and segmental duplication were analyzed. It was found that there were 71 pairs of gene duplications in the alfalfa *SPL* gene family (Supplementary Table S6), including *MsSPL15-2*/*MsSPL15-5* and *MsSPL15-2*/*MsSPL15-3* two pairs of tandem duplicatiors (Table 3), *MsSPL1-1*/*MsSPL1-2* and other 69 pairs of segmental duplicators.

**Table 3:** Duplicated *MsSPL* genes and the divergence time of *MsSPL* genes.

Gene 1	Gene 2	Ka	Ks	Ka/Ks	MYA	Duplication type
*MsSPL13-1*	*MsSPL13-4*	0.008	0.007	1.217	0.450	Segmental
*MsSPL15-2*	*MsSPL15-5*	0.139	0.372	0.373	24.824	Tandem
*MsSPL15-2*	*MsSPL15-3*	0.139	0.372	0.373	24.824	Tandem
*MsSPL15-3*	*MsSPL13-4*	0.005	0.004	1.097	0.274	Segmental
*MsSPL15-5*	*MsSPL13-4*	0.005	0.005	1.105	0.303	Segmental

The Ka/Ks (non-synonymous/synonymous) value is widely used to represent gene selection pressure and evolutionary rate: a Ka/Ks value greater than 1 indicates evolutionary accelerated positive selection, and Ka/Ks = 1 indicates neutrality gene drift, Ka/Ks < 1 indicates purification selection under functional constraints. To further analyze the selection pressure of the alfalfa *SPL* gene family, the non-synonymous substitution rate Ka, synonymous substitution rate Ks, and Ka/Ks ratio of 71 pairs of homologous genes were analyzed (Supplementary Table S6). The homologous gene Ka/Ks ratio is < 1, ranging from 0.042 (*MsSPL8-3/8-4*) to 0.844 (*MsSPL13-2/13-4*, *MsSPL13-3/13-4*), indicating that the alfalfa *SPL* gene has undergone a great purification selection pressure so that its function can be maintained. It reflected that they do not have much functional differentiation in the evolution process and are highly conserved. The Ka/Ks ratio of the three pairs of homologs, *MsSPL13-1/13-4*, *MsSPL13-3/13-4*, and *MsSPL15-5/13-4*, was > 1, indicating that they had undergone positive evolutionary selection, and these three pairs of genes may be responsible for differentiating new functions (Table 3).

The ancestors of the legume family originated about 67 million years ago, whereas the divergence time of Papilionoideae Subfamily, which includs the genus alfalfa, was approximately 34–63.7 MYA. The evolutionary divergence time (millions of years, MYA) between the genes of alfalfa *SPLs* was calculated using the formula T = Ks/2λ × 10^–6^ (λ = 6.5 × 10^–9^). The results showed that *MsSPL3-1/17-2*, *MsSPL3-1/17-1*, *MsSPL3-2/17-2*, *MsSPL3-2/17-1*, *MsSPL3-4/17-1*, *MsSPL3-4/17-2*, *MsSPL3-3/17-1*, *MsSPL3-3/17-2*, *MsSPL17-4/3-1*, *MsSPL17-4/3-2*, *MsSPL17-4/3-4*, *MsSPL17-4/3-3* (Supplementary Table S6)*,* which the 12 homologous gene pairs were derived from the formation period of Papilionoideae Subfamily (about 42 MYA). Three pairs of *MsSPL15-2/15-5*, *MsSPL15-2/15-3*, and *MsSPL15-2/14* homologous gene pairs were derived 24.8 million years ago. The remaining homologous gene pairs underwent gene duplication between 0.214 (*MsSPL21-1/21-3*, *MsSPL21-1/21-2*) and 6.511 (*MsSPL19-3/19-1*) million years ago.

### Expression analysis of alfalfa *MsSPL* genes under drought stress

**Figure 6:**
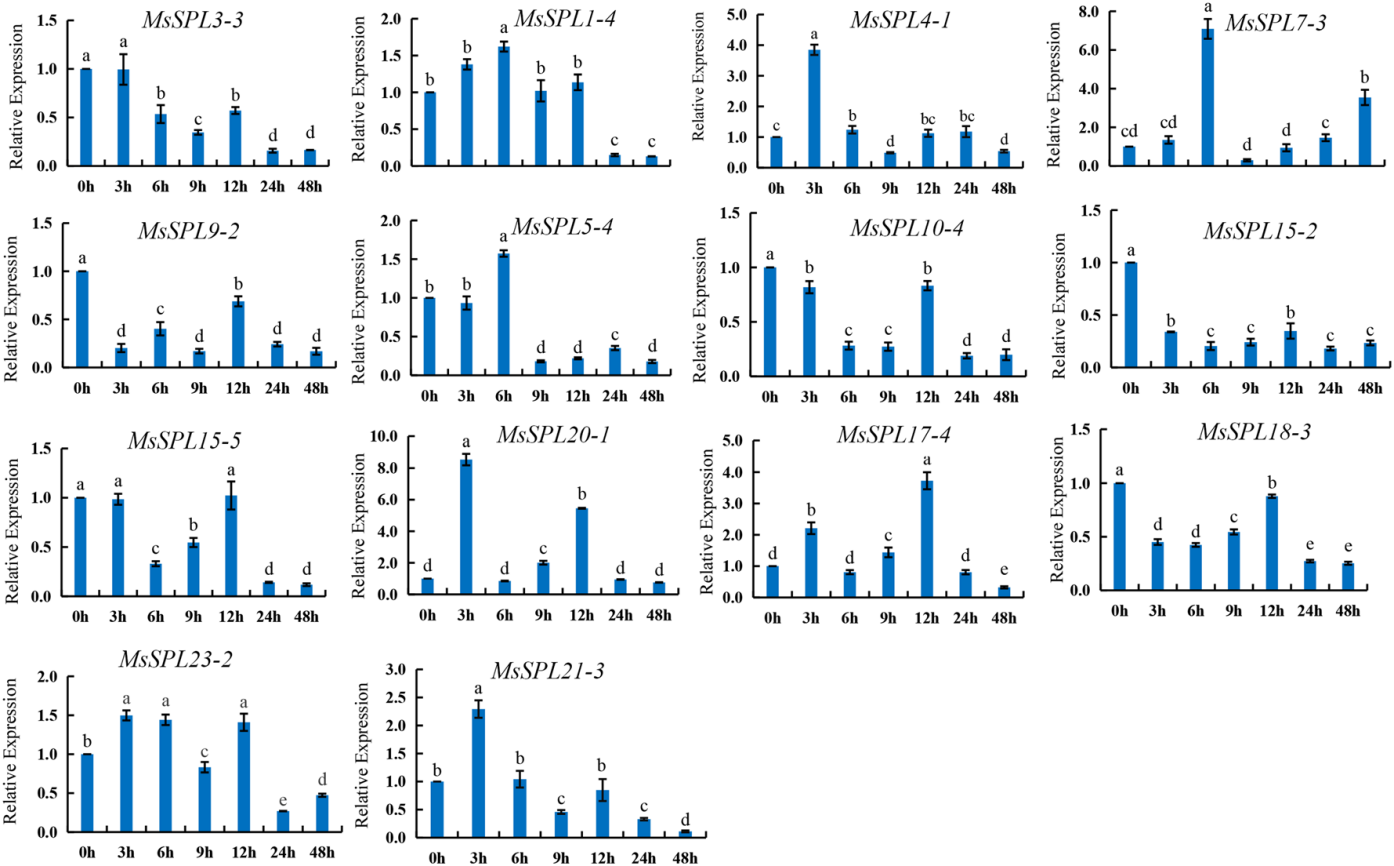
Gene expression of 14 *MsSPL* genes under drought treatment at 0 h, 3 h, 6 h, 9 h, 12 h, 24 h and 48 h was analyzed using qRT-PCR. Error bars represent standard errors of three biological replicates. The differernt lowercase letters indicate significant differences at the *P* < 0.05 level.

In order to clarify the expression patterns of the alfalfa *SPL* gene family under drought stress, qRT-PCR was used to analyze the expression patterns of *MsSPL3-3*, *MsSPL1-4*, *MsSPL4-1*, *MsSPL7-3*, *MsSPL9-2*, *MsSPL5-4*, *MsSPL10-4*, *MsSPL15-2*, *MsSPL15-5*, *MsSPL20-1*, *MsSPL17-4*, *MsSPL18-3*, *MsSPL23-2*, and *MsSPL21-3* under drought stress (Fig. 6). The analysis showed that under drought stress, compared with the control (0 h), *MsSPL4-1*, *MsSPL20-1*, *MsSPL17-4*, *MsSPL23-2*, and *MsSPL21-3* were significantly up-regulated at 3 h treatment, and *MsSPL1-4*, *MsSPL7-3*, *MsSPL5-4*, and *MsSPL23-2* were significantly increased after 6 h under drought treatment. It can be seen that the *SPL* genes positively responds to drought stress in alfalfa. The time points of *MsSPL9-2*, *MsSPL10-4*, *MsSPL15-2*, and *MsSPL18-3* under drought stress were all lower than those of the control, *MsSPL3-3* was under drought stress for 3 h, and *MsSPL15-5* was not significantly different from the control at 3 h and 6 h, and the rest of the time was lower than the control treatment. It indicated that these 6 genes had an obvious response to drought stress.

### Expression analysis of alfalfa *MsSPL* genes under salt stress

**Figure 7:**
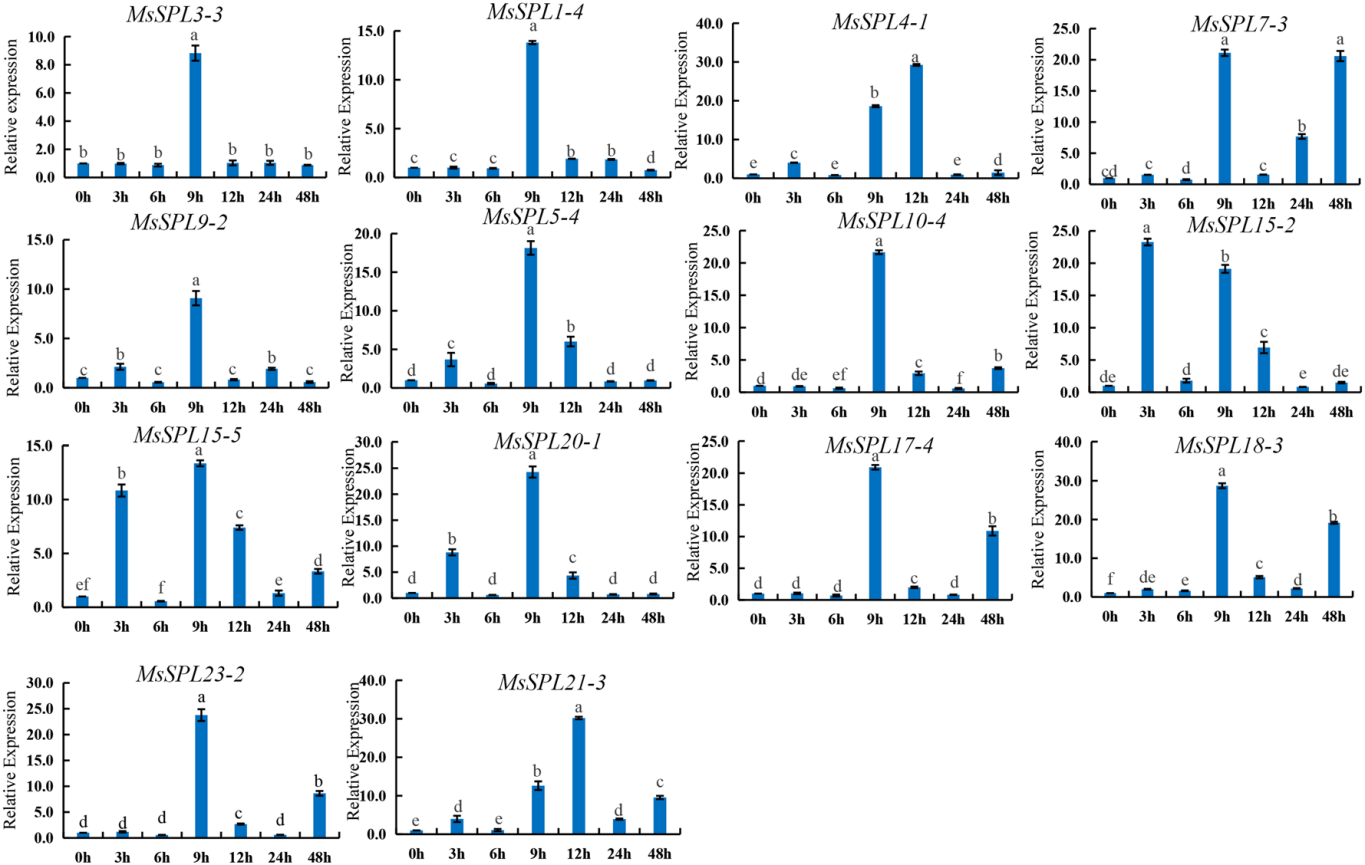
Gene expression of 14 *MsSPL* genes under NaCl treatment at 0 h, 3 h, 6 h, 9 h, 12 h, 24 h and 48 h was analyzed using qRT-PCR. Error bars represent standard errors of three biological replicates. The differernt lowercase letters indicate significant differences at the *P* < 0.05 level.

To further reveal the potential function of the *MsSPL* genes in abiotic salt stress (NaCl), the expression levels of 14 genes, including *MsSPL3-3*, *MsSPL1-4*, *MsSPL4-1*, *MsSPL7-3* et al., were analyzed by qRT-PCR (Fig. 7). *MsSPL3-3*, *MsSPL1-4*, *MsSPL9-2*, *MsSPL5-4*, *MsSPL10-4*, *MsSPL15-5*, *MsSPL20-1*, *MsSPL17-4*, *MsSPL18-3*, and *MsSPL23-2* all reached the most significant level after 9 h of NaCl treatment, among which *MsSPL10-4*, *MsSPL17-4*, *MsSPL18-3*, and *MsSPL23-2* had the same expression pattern, showing a trend of first increasing, then decreasing and then increasing. *MsSPL4-1* and *MsSPL21-3* reached the maximum at 12 h of treatment and *MsSPL7-3* reached the highest value at 9 h and 48 h of treatment, indicating that these genes actively responded to salt stress.

### Expression pattern analysis of *MsSPL* genes in response to Me JA

**Figure 8:**
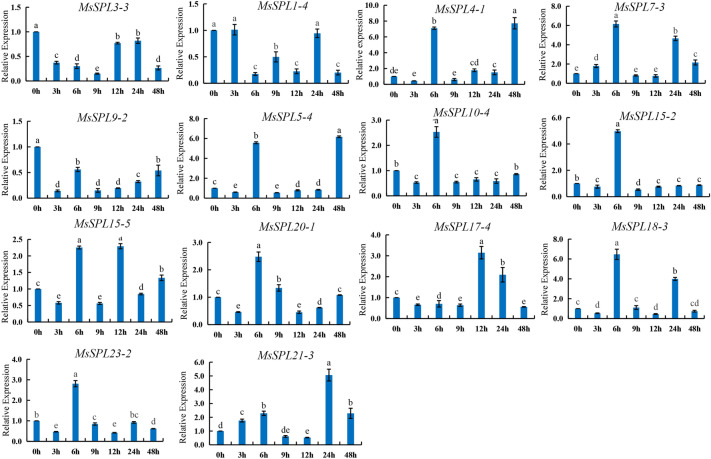
Gene expression of 14 *MsSPL* genes under Me JA treatment at 0 h, 3 h, 6 h, 9 h, 12 h, 24 h and 48 h was analyzed using qRT-PCR. Error bars represent standard errors of three biological replicates. The differernt lowercase letters indicate significant differences at the *P* < 0.05 level.

Real-time quantitative PCR was used to detect the relative expression of the *MsSPL* genes after alfalfa was sprayed with Me JA for 0 h, 3 h, 6 h, 9 h, 12 h, 24 h, and 48 h. The results were shown in Fig. 8, *MsSPL4-1*, *MsSPL7-3*, *MsSPL5-4*, *MsSPL10-4*, *MsSPL15-2*, *MsSPL15-5*, *MsSPL20-1*, *MsSPL18-3*, and *MsSPL23-2* were significantly increased after Me JA spraying for 6 h, *MsSPL17-4* reached the highest after 12 h, and *MsSPL21-3* reached the highest at 24 h, indicating that methyl jasmonate can induce alfalfa differential expression of *MsSPL* genes. *MsSPL3-3* and *MsSPL9-2* were significantly lower than the control at each time point, showing negative regulation.

## Discussion

As a plant-specific transcription factor, SPL has a highly conserved SBP domain. *SPL* gene family has been identified in Arabidopsis (16)^1^, Wheat (56)^5^, Mustard greens (59)^28^, Populus euphratica (32)^8^, Pecan (32)^29^, Soybean (41)^30^ and other species. In this study, 82 SPL sequences were identified by bioinformatics methods. These sequences were divided into 23 genes containing 4 (30.43%), 3 (43.47%), 2 (13.04%) and 1 (13.04%) alleles (Table 1). As a type of transcription factor unique to higher plants, the expression product of SPLs should be located in the nucleus and play a regulatory role in the expression of downstream genes. The subcellular localization predictions showed that all MsSPL proteins were located in the nucleus. The results were consistent with the subcellular localization results of *SPL* gene family in Arabidopsis^4^. This suggests that SPL may function as a nuclear transcription factor.

Analysis of SPL evolution in different species helps to predict gene function. By protein multiple sequence alignment and phylogenetic tree analysis, the identified 23 MsSPL proteins were classified into six subfamilies with the SPL proteins of *A. thaliana* (16) and *M. truncatula* (28) (Fig. 2), each subgroup from the *A. thaliana*, *M. truncatula,* and *M. sativa* have an unequal number of proteins, with 19 orthologs including MsSPL9 and MtSPL04, MsSPL7 and MtSPL06, MsSPL1 and MtSPL01 et al., and 9 paralogs pairs are AtSPL02 and AtSPL03, AtSPL04 and AtSPL05, which indicated that the MsSPLs is closely related to MtSPLs. The function of the gene is closely related to its structure. Analysis of gene structure and conserved motifs showed that* MsSPL* alleles and paralogs have similar motifs and exons/introns, which not only verified the construction of phylogenetic tree, but also further supported the conserved evolutionary characteristics of *SPL* gene family. 10 out of 82 alleles and paralogs contained no intron, and 72 contained one or more introns (Fig. 3). Previous studies reviewed that lacking introns in the coding regions is gengeally associated with rapid changing expression levels during cell division and cell differentiation, contributing to the generation of a new generation genes^31^.

Chromosome location analysis showed that 82 MsSPL sequences were unevenly distributed on 26 chromosomes (Fig. 1). Segmental duplication and tandem duplication were the main driving forces for gene acquisition of new functions and family expansion^32^. Among them, *MsSPL15-2/MsSPL 15-5*, *MsSPL15-2/MsSPL15-3,* two pairs of tandem duplication genes (Table 3), and 69 pairs of segmental duplication genes, indicating that segmental duplication is the main driving force for the evolutionary expansion of the *MsSPL* gene family. New research showed that angiosperms also had a whole genome duplication event 20 million years ago^33^. The analysis showed that the duplication time of the three pairs of genes, *MsSPL15-2/15-5*, *MsSPL15-2/15-3*, and *MsSPL15-2/14*, coincided with the time of the whole genome duplication event. The Ka/Ks ratio of 96% of homologous genes was less than 1 (Supplementary Table S6), indicating that purification selection played an important role in the evolution of SPL transcription factors in alfalfa and was highly conserved.

Using protein network interactions to connect proteins of unknown function to protein interaction networks will contribute to further understanding of protein biological functions enriched by protein network interactions, and dynamic regulatory networks between various biological logic molecules in cells^34^^,^^35^. Therefore, it is necessary to predict the potential interacting proteins of MsSPL proteins and their functions. AES82580 is a superoxide dismutase protein, *MsSPL8* interact with AES82580 (Fig. 5), indicating that these proteins may be involved in scavenging free radicals and repairing related damaged cells. Plastocyanin is a class of copper-containing electron transfer proteins involved in plant photosynthesis, growth and development, and adaptation to the environment^36^. *MsSPL8* also interact with plastocyanin, suggesting that these proteins may be related to plastocyanin. LFY is a unique transcription factor in plants. It acts as the downstream of auxin response factor to promote the initiation of flower primordia and the formation of flower organs^37^^,^^38^. Our research analysis showed that multiple MsSPLs interact with LFY transcription factors. This is consistent with Gao et al.^28^^,^^39^^,^^40^ that SPLs induce expression in floral organs.

A growing number of studies have shown that SPL transcription factors play an important regulatory role in inducing plant development and participating in plant biological functions. SPLs were found to be involved in wheat spike development in wheat^5^. SPLs are involved in early anther development in *A. thaliana*^4^. Citrus *SPL* gene was able to promote *A. thaliana* flowering independently of photoperiod^41^. The *MtmiR156/MtSPL* module in *M. truncatula* is involved in developing leaves, branches, and seed pods^42^. Most *BpSPL* genes in *Betula platyphylla* had high transcription levels in leaves, female inflorescences, and male inflorescences^12^. Most studies have shown that SPL transcription factors play an important role in responding to biotic and abiotic stresses. In lychee, 10 *LcSPLs* were highly expressed in cold response, and only *LcSPL1* and *LcSPL2* were associated with age-dependent flowering in response to cold^43^. The adaptability to salt and drought stress environments was improved in birch trees overexpressing *BpSPL9*^44^. In response to exogenous hormone treatment, including indoleacetic acid (IAA), gibberellic acid (GA3), methyl jasmonic acid (Me JA), and abscisic acid (ABA), *SmSPL6* exhibited different expression patterns^45^. Studies have shown that cis-acting elements in the promoter region have an impact on the number, type, and distribution of gene expression in different regulatory roles^46–48^. Therefore, the analysis of promoter regulatory elements is essential to study the function of specific genes. A large number of auxin-responsive elements, salicylic acid cis-acting elements, methyl jasmonate-responsive elements, abscisic acid-responsive elements, gibberellin cis-acting elements, and elements involved in abiotic stress response were predicted in the promoter region of *MsSPL* genes (Fig. 4). These include defense and stress, low-temperature, wound response, and drought response elements that play key roles in plant responses to biotic and abiotic stresses, as well as plant signal transduction. Previous studies mainly focused on the role of *SPL* genes in growth and development. In contrast, few studies on the function of *SPL* genes under stress, drought, salt, and methyl jasmonate stress treatment of alfalfa, the results of qRT-PCR showed that under drought stress, *MsSPL1-4/4-1/7-3/5-4/23-2/21-3* contain ABA response elements, and these genes are significantly up-regulated under drought stress, indicating that may belong to the abscisic acid-dependent pathway; *MsSPL9-2/18-3* do not contain drought response elements, and the genes are down-regulated, *MsSPL20-1/17-4* contained drought-responsive elements, and gene expression was up-regulated, indicating that the drought-responsive elements of these four genes may be positively correlated with gene expression (Fig. 6). Studies have shown that high-salt environment can accelerate the accumulation of ABA in plants, and ABA accumulation can induce the expression of ABA-responsive element genes, thereby making plants resistant. In our study, 12 *MsSPLs* contained ABA-responsive elements, and all 12 genes tended to be up-regulated under salt stress (Fig. 7). This is consistent with the study of Irfan^49^. It is worth noting that the *MsSPL3-3* contains five methyl jasmonate response elements. Under Me JA treatment, the *MsSPL3-3* was significantly down-regulated, and the *MsSPL4-1/7-3/17-4/18-3/23-2/21-3* without the methyl jasmonate response element were significantly up-regulated. (Fig. 8), which indicated that the methyl jasmonate response element in the promoter region was negatively correlated with possible gene expression. This indicated that *MsSPL* genes actively respond to biotic and abiotic stresses.

## Conclusion

A detailed analysis of the phylogeny, gene structure, conserved motifs, protein interactions, cis-acting elements, and expression profiles of members of the *MsSPL* gene family was carried out. A total of 23 *MsSPL* genes were identified from the alfalfa genome in this study. Together with *A. thaliana* and *M. truncatula* to construct a phylogenetic tree, the SPL proteins can be divided into six subgroups. There are similar gene structures and conserved motifs in the same subgroup, and they are more closely related to *M. truncatula*. Segmental duplication is the main form of gene family expansion, and purifying selection plays an major role in the evolution of SPL transcription factors in alfalfa. Analysis of cis-acting elements revealed that *MsSPLs* are regulated by plant hormones and various stresses. QRT-PCR analysis showed that the *MsSPL* gene family had different spatiotemporal expression patterns under drought stress, salt stress, and methyl jasmonate stress. Collectively, our data add to the understanding of the genetic evolutionary relationships and biological functions of *MsSPLs*. Ultimately, the results of this study lay the foundation for further revealing the functional characteristics of the *SPL* gene family.

## Materials and methods

### Plant material and stress treatments

The seeds of alfalfa variety "Sundeli" provided by Jiuquan Future Grass Industry Co., LTD. (scientific name *M.sativa* L., variety registration No. 247, variety type introduction) were selected. Alfalfa seeds were cultivated in soil with a 3:1 ratio of vegetative soil to vermiculite. It was then placed in a tissue culture chamber with a photoperiod of 12 h darkness /12 h light and a temperature of 25 ± 1 °C for growth. During the experiment, the alfalfa was irrigated regularly with 1/10 Hoagland nutrient solution to prevent malnutrition. At the age of 4 weeks, the seedlings were irrigated with 10% PEG-6000, 100 mmol/L NaCl and sprayed with 200 mmol/L methyl jasmonate, 6 copies for each treatment. The samples were collected at 0 h, 3 h, 6 h, 9 h, 12 h, 24 h and 48 h, respectively, and then placed in – 80 °C cryopreservation for subsequent quantitative experiments.

### RNA extraction, reverse transcription, and real-time quantitative PCR

After sample collection, total RNA was extracted using a UNIQ-10 column Trizol total RNA extraction kit (Sangon Bioengineering Co., Ltd., Shanghai), and the total RNA was extracted using TIANScript II RT Kit kit (Tiangen Biochemical Technology Co., Ltd., Beijing). Transcribed into cDNA. NCBI Premier-BLAST was used to design primers (Supplementary Table S5), the amplified fragments were about 100–200 bp, alfalfa *GAPDH* was used as the internal reference, and the Quantageneq 225 real-time PCR instrument was used to complete the entire experiment. The experimental reaction system (20 μL) was: 10 μL 2 × ChamQ Universal SYBR qPCR Master Mix (Vazyme), 1 μL cDNA, 1 μL upstream and downstream primers, 7 μL ddH_2_O. The above reaction components were added, centrifuged, mixed, and then placed in a real-time fluorescence quantitative PCR instrument for amplification. After the reaction is complete, the specificity of the PCR product is determined by examining the melting curve. Each reaction was repeated three times, and 2^-ΔΔCT^ was used to calculate the relative expression of genes under different treatment times. Each experiment was repeated three times with independent RNA sample. The raw data of qRT-PCR are shown in Supplementary Table S4.

### Statistical analysis

Excel 2010 was used for Statistics and calculation of relevant data, SPSS Statistics 19.0 software was used for analysis of variance, and student's t-test was used to compare the mean values at 5% significance level. The results are the average of three repeated experiments.

### Identification of *SPL* gene family members

Download the SPL protein sequences of *A. thaliana* and *M. truncatula* from the Phytozome database (https://phytozome.jgi.doe.gov/pz/portal.html). The alfalfa genome data, CDS sequences, and protein sequences used in this experiment were downloaded at (https://figshare.com/projects/whole_genome_sequencing_and_assembly_of_Medicago_sativa/66380). The *A. thaliana* SPL sequence was used as the query sequence, and BLAST was used to identify the alfalfa genome. Then use Pfam (http://pfam.xfam.org/)^50^ and the SMART website (http://smart.embl-heidelberg.de/)^51^ to predict the structure of the sequence obtained in the previous step, knock out sequences that do not contain the typical SBP binding domain of MsSPL proteins, the rest of the protein sequence is regarded as a member of the MsSPL family.

### Physicochemical property analysis and chromosomal location analysis of alfalfa *SPL*

MsSPL amino acid residues, overall average hydrophilicity, isoelectric point, and molecular weight were analyzed using the ExPaSy protein server (https://web.expasy.org/protparam/)^52^. WoLF PSORT (https://wolfpsort.hgc.jp/)^53^ was used to predict the subcellular location of MsSPL proteins. The location information of all *MsSPL* genes in the alfalfa genome was extracted, and the online tool TBtools was used to map the *MsSPL* genes to the corresponding chromosomes.

### Phylogenetic classification analysis

Based on multiple sequence alignments of MsSPL, MtSPL and AtSPL proteins could be divided into various groups. We performed phylogenetic analysis with MEGA 7.0^54^. The phylogenetic tree image was enhanced by the Evolview online program (http://www.evolgenius.info/evolview)^55^.

### Gene structure and conserved motif analysis

The online tool GSDS 2.0 (http://gsds.cbi.pku.edu.cn/)^56^ was used to analyze the gene structure of the *MsSPL* gene family. Protein conserved domains by MEME (http://meme-suite.org/tools/meme)^57^; the maximum number of motif is 10, motifs containing 10-50 amino acids and E-values < 1e^−20^ are characterized and published in MsSPL comparisons are made between genes to identify group-specific or group-conserved signatures.

### Analysis of cis-acting elements

The upstream sequence (1 ~ 2000 bp) of the *MsSPL* gene was extracted from the alfalfa database using TBtools software, and the cis-form in its promoter was identified by PlantCARE (http://bioinformatics.psb.ugent.be/webtools/plantcare/html/)^58^. Elements of action were visualized and analyzed with TBtools software.

### Construction of protein interaction network

To predict the protein–protein interaction network of the alfalfa SPL family, the alfalfa SPL protein interaction network was constructed using the STRING online website (https://string-db.org/)^59^.

### Analysis of gene duplication and divergence time

Using NCBI-Protein Blast to align all amino acid sequences of the alfalfa *SPL* gene online and screen genes with a coverage rate of > 75% and identity of > 75%, this pair of genes is regarded as a duplicated gene. In addition, in a 100 kb region, two genes separated from multiple genes are considered tandem repeats^60^. The "Simple Ka/Ks Calculator" in TBtools (https://github.com/CJ-Chen/TBtools) was used to calculate the nonsynonymous (Ka) and synonymous (Ks) substitution rates of gene duplication pairs. The ratio of Ka/Ks to judge the selection pressure of replicating genes. Ka/Ks < 1 means purification selection, Ka/Ks = 1 means neutral selection, and Ka/Ks > 1 means positive selection^61^. The divergence time between *SPL* gene pairs is expressed in million years ago (MYA), and the calculation formula for divergence time is T = Ks/2λ (λ = 6.5 × 10^–9^), where λ represents each synonym per year Synonymous mutation substitution rate for points.

### Ethics approval and consent to participate

This study does not include human or animal subjects.

### Statement on guidelines

All experimental studies and experimental materials involved in this research are in full compliance with relevant institutional, national, and international guidelines and legislation.

## Supplementary Information

Supplementary Information 1.

Supplementary Information 2.

Supplementary Information 3.

Supplementary Information 4.

Supplementary Information 5.

Supplementary Information 6.

Supplementary Information 7.

## Data Availability

The alfalfa genome data, CDS sequences, and protein sequences used in this experiment were downloaded at (https://figshare.com/projects/whole_genome_sequencing_and_assembly_of_Medicago_sativa/66380), and the SPL protein sequences of *A. thaliana* and *M. truncatula* were downloaded from the Phytozome database (https://phytozome.jgi.doe.gov/pz/portal.html). The original contributions presented in this study are included in the article/supplementary material, further inquiries can be directed to the corresponding authors.
