# Supplementary figures and images for: Identification of Alfalfa SPL gene family and expression analysis under biotic and abiotic stresses
Source: Sci Rep. 2023 Jan 3;13:84. doi: 10.1038/s41598-022-26911-7 (PMC9810616; doi:10.1038/s41598-022-26911-7)

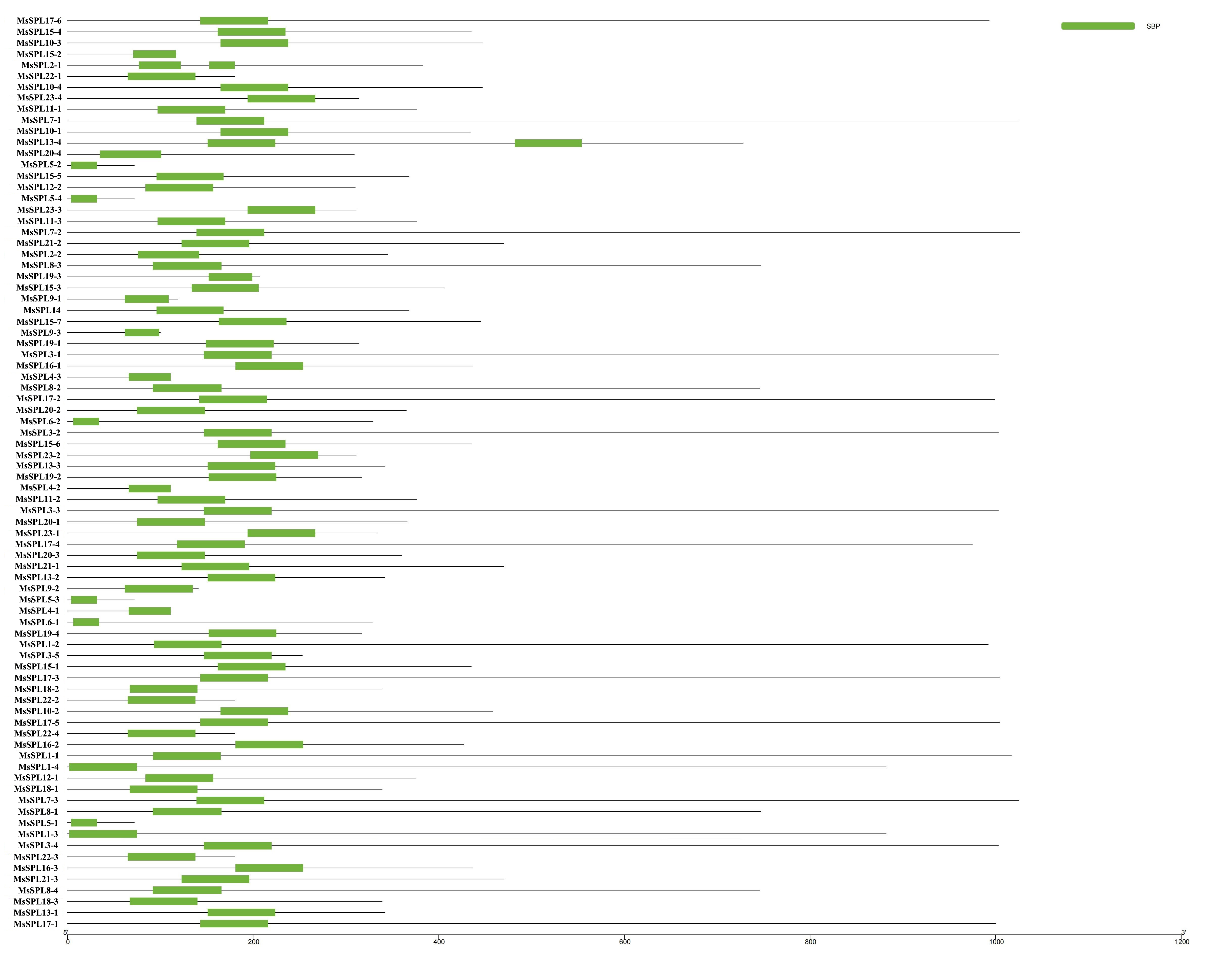


**Supplementary Fig. 1** Binding domain analysis of MsSPL proteins family

Supplement: Supplementary file 1 — Supplementary Information 1. [file 41598_2022_26911_MOESM1_ESM.docx]
