# Supplementary material for: Identification of Alfalfa SPL gene family and expression analysis under biotic and abiotic stresses
Source: Sci Rep. 2023 Jan 3;13:84. doi: 10.1038/s41598-022-26911-7 (PMC9810616; doi:10.1038/s41598-022-26911-7)
Supplement: Supplementary file 2 — Supplementary Information 2. [file 41598_2022_26911_MOESM2_ESM.docx]

**Supplementary Table 1** Basic physical and chemical properties of *MsSPLs.*

| Gene ID | Gene | Gene location | | Size (aa) | Molecular weight (D) | Isoelectric point | GRAVY | Subcellular Localization |
| --- | --- | --- | --- | --- | --- | --- | --- | --- |
| MS.gene026366.t1 | *MsSPL1-1* | 19452387 | 19457960 | 1017 | 113299.24 | 7.74 | -0.558 | nucleus |
| MS.gene040800.t1 | *MsSPL1-2* | 19309840 | 19315235 | 992 | 110419.89 | 7.53 | -0.571 | nucleus |
| MS.gene061710.t1 | *MsSPL1-3* | 19418650 | 19423753 | 882 | 97811.86 | 7.30 | -0.564 | nucleus |
| MS.gene061104.t1 | *MsSPL1-4* | 19351715 | 19356819 | 882 | 97842.98 | 7.54 | -0.558 | nucleus |
| MS.gene97631.t1 | *MsSPL2-1* | 40115888 | 40120381 | 383 | 41937.71 | 9.23 | -0.506 | nucleus |
| MS.gene018772.t1 | *MsSPL2-2* | 37663643 | 37667677 | 345 | 37697.81 | 9.37 | -0.659 | nucleus |
| MS.gene005475.t1 | *MsSPL3-1* | 64107643 | 64114964 | 1003 | 111834.69 | 5.96 | -0.481 | nucleus |
| MS.gene07353.t1 | *MsSPL3-2* | 64933391 | 64940736 | 1003 | 111747.50 | 5.91 | -0.479 | nucleus |
| MS.gene20896.t1 | *MsSPL3-3* | 61116783 | 61124128 | 1003 | 111747.50 | 5.91 | -0.479 | nucleus |
| MS.gene050360.t1 | *MsSPL3-4* | 64962573 | 64969918 | 1003 | 111733.48 | 5.91 | -0.479 | nucleus |
| MS.gene050361.t1 | *MsSPL3-5* | 64974675 | 64976750 | 253 | 28035.25 | 9.26 | -0.776 | nucleus |
| MS.gene060699.t1 | *MsSPL4-1* | 21706562 | 21706896 | 111 | 12625.24 | 6.37 | -0.731 | nucleus |
| MS.gene71881.t1 | *MsSPL4-2* | 15830531 | 15830865 | 111 | 12625.24 | 6.37 | -0.731 | nucleus |
| MS.gene58356.t1 | *MsSPL4-3* | 19045183 | 19045517 | 111 | 12625.24 | 6.37 | -0.731 | nucleus |
| MS.gene060698.t1 | *MsSPL5-1* | 21709268 | 21709486 | 72 | 8447.27 | 10.60 | -1.347 | nucleus |
| MS.gene71880.t1 | *MsSPL5-2* | 15833176 | 15833394 | 72 | 8447.27 | 10.60 | -1.347 | nucleus |
| MS.gene58357.t1 | *MsSPL5-3* | 19047823 | 19048041 | 72 | 8447.27 | 10.60 | -1.347 | nucleus |
| MS.gene059775.t1 | *MsSPL5-4* | 19691056 | 19691274 | 72 | 8438.26 | 10.60 | -1.351 | nucleus |
| MS.gene77091.t1 | *MsSPL6-1* | 32885447 | 32887855 | 329 | 35815.66 | 6.35 | -0.472 | nucleus |
| MS.gene77090.t1 | *MsSPL6-2* | 32952303 | 32954711 | 329 | 35815.66 | 6.35 | -0.472 | nucleus |
| MS.gene98509.t1 | *MsSPL7-1* | 44943312 | 44951517 | 1025 | 113039.31 | 6.72 | -0.356 | nucleus |
| MS.gene033954.t1 | *MsSPL7-2* | 40371650 | 40380096 | 1026 | 113111.59 | 6.82 | -0.338 | nucleus |
| MS.gene062030.t1 | *MsSPL7-3* | 44103168 | 44111298 | 1025 | 113015.41 | 6.95 | -0.359 | nucleus |
| MS.gene002799.t1 | *MsSPL8-1* | 66919581 | 66926272 | 747 | 83747.01 | 6.35 | -0.263 | nucleus |
| MS.gene96180.t1 | *MsSPL8-2* | 64605071 | 64612074 | 746 | 83681.92 | 6.35 | -0.273 | nucleus |
| MS.gene00967.t1 | *MsSPL8-3* | 66634515 | 66641207 | 747 | 83767.08 | 6.35 | -0.262 | nucleus |
| MS.gene004483.t1 | *MsSPL8-4* | 66422276 | 66428967 | 746 | 83637.86 | 6.35 | -0.272 | nucleus |
| MS.gene002482.t1 | *MsSPL9-1* | 70786845 | 70787204 | 119 | 13759.13 | 5.72 | -1.053 | nucleus |
| MS.gene047335.t1 | *MsSPL9-2* | 70573344 | 70575469 | 141 | 16578.21 | 6.71 | -1.294 | nucleus |
| MS.gene01060.t1 | *MsSPL9-3* | 70708782 | 70709083 | 100 | 11483.54 | 5.33 | -1.161 | nucleus |
| MS.gene75620.t1 | *MsSPL10-1* | 71181850 | 71183912 | 434 | 48015.19 | 8.71 | -0.832 | nucleus |
| MS.gene77585.t1 | *MsSPL10-2* | 79495037 | 79497099 | 458 | 50713.43 | 8.67 | -0.772 | nucleus |
| MS.gene045290.t1 | *MsSPL10-3* | 79517454 | 79519516 | 447 | 49620.04 | 8.71 | -0.828 | nucleus |
| MS.gene045288.t1 | *MsSPL10-4* | 79530419 | 79532481 | 447 | 49577.01 | 8.59 | -0.808 | nucleus |
| MS.gene06231.t1 | *MsSPL11-1* | 78961642 | 78965165 | 376 | 41668.35 | 6.56 | -0.679 | nucleus |
| MS.gene055507.t1 | *MsSPL11-2* | 82451931 | 82456700 | 376 | 41642.36 | 6.62 | -0.655 | nucleus |
| MS.gene013155.t1 | *MsSPL11-3* | 81624631 | 81629400 | 376 | 41642.36 | 6.62 | -0.655 | nucleus |
| MS.gene030453.t1 | *MsSPL12-1* | 6758141 | 6761259 | 375 | 41507.81 | 8.78 | -0.788 | nucleus |
| MS.gene95236.t1 | *MsSPL12-2* | 126713 | 129633 | 310 | 34566.43 | 9.41 | -0.838 | nucleus |
| MS.gene09220.t1 | *MsSPL13-1* | 15072371 | 15074623 | 342 | 38332.61 | 8.68 | -0.796 | nucleus |
| MS.gene023426.t1 | *MsSPL13-2* | 14058864 | 14061105 | 342 | 38293.62 | 8.89 | -0.785 | nucleus |
| MS.gene08539.t1 | *MsSPL13-3* | 14099838 | 14102079 | 342 | 38293.62 | 8.89 | -0.785 | nucleus |
| MS.gene09027.t1 | *MsSPL13-4* | 15375680 | 15382659 | 728 | 82273.72 | 8.60 | -0.775 | nucleus |
| MS.gene09219.t1 | *MsSPL14* | 15077272 | 15079598 | 368 | 41717.46 | 7.95 | -0.767 | nucleus |
| MS.gene09218.t1 | *MsSPL15-1* | 15082163 | 15084677 | 435 | 48597.99 | 5.36 | -0.627 | nucleus |
| MS.gene023428.t1 | *MsSPL15-2* | 14032506 | 14032859 | 117 | 13110.04 | 9.13 | -0.504 | nucleus |
| MS.gene023425.t1 | *MsSPL15-3* | 14063788 | 14065985 | 406 | 46025.30 | 6.94 | -0.768 | nucleus |
| MS.gene023424.t1 | *MsSPL15-4* | 14068549 | 14071063 | 435 | 48561.94 | 5.43 | -0.633 | nucleus |
| MS.gene08540.t1 | *MsSPL15-5* | 14104689 | 14106772 | 368 | 41714.46 | 7.95 | -0.773 | nucleus |
| MS.gene08541.t1 | *MsSPL15-6* | 14109341 | 14111855 | 435 | 48573.99 | 5.43 | -0.621 | nucleus |
| MS.gene09028.t1 | *MsSPL15-7* | 15385042 | 15387587 | 445 | 49564.98 | 5.55 | -0.637 | nucleus |
| MS.gene071085.t1 | *MsSPL16-1* | 34524923 | 34529031 | 437 | 49474.94 | 6.15 | -0.707 | nucleus |
| MS.gene054760.t1 | *MsSPL16-2* | 33893126 | 33897220 | 427 | 48303.67 | 6.42 | -0.722 | nucleus |
| MS.gene029630.t1 | *MsSPL16-3* | 36619037 | 36623140 | 437 | 49440.93 | 6.11 | -0.706 | nucleus |
| MS.gene050425.t1 | *MsSPL17-1* | 4468226 | 4476562 | 1000 | 110751.84 | 5.94 | -0.404 | nucleus |
| MS.gene28650.t1 | *MsSPL17-2* | 4941652 | 4949961 | 999 | 110681.78 | 5.78 | -0.393 | nucleus |
| MS.gene050856.t1 | *MsSPL17-3* | 4907441 | 4915856 | 1004 | 111023.29 | 6.01 | -0.390 | nucleus |
| MS.gene072658.t1 | *MsSPL17-4* | 4933784 | 4942117 | 975 | 107901.51 | 5.98 | -0.407 | nucleus |
| MS.gene072657.t1 | *MsSPL17-5* | 5356845 | 5365260 | 1004 | 111023.29 | 6.01 | -0.390 | nucleus |
| MS.gene050857.t1 | *MsSPL17-6* | 2632 | 11041 | 993 | 109846.01 | 6.01 | -0.388 | nucleus |
| MS.gene017969.t1 | *MsSPL18-1* | 14588366 | 14591721 | 339 | 36694.52 | 9.06 | -0.694 | nucleus |
| MS.gene04608.t1 | *MsSPL18-2* | 15663771 | 15667121 | 339 | 36668.44 | 9.06 | -0.707 | nucleus |
| MS.gene04607.t1 | *MsSPL18-3* | 15702310 | 15705660 | 339 | 36668.44 | 9.06 | -0.707 | nucleus |
| MS.gene36181.t1 | *MsSPL19-1* | 55277097 | 55281018 | 314 | 34684.42 | 8.80 | -0.725 | nucleus |
| MS.gene010796.t1 | *MsSPL19-2* | 55920343 | 55924440 | 317 | 34819.58 | 8.94 | -0.713 | nucleus |
| MS.gene38329.t1 | *MsSPL19-3* | 57646643 | 57647266 | 207 | 22123.72 | 8.81 | -0.499 | nucleus |
| MS.gene93246.t1 | *MsSPL19-4* | 57989999 | 57993969 | 317 | 34858.68 | 8.90 | -0.697 | nucleus |
| MS.gene000446.t1 | *MsSPL20-1* | 68936082 | 68938686 | 366 | 40921.78 | 8.80 | -0.857 | nucleus |
| MS.gene99828.t1 | *MsSPL20-2* | 71401487 | 71404046 | 365 | 40708.53 | 8.83 | -0.839 | nucleus |
| MS.gene001948.t1 | *MsSPL20-3* | 72048323 | 72050879 | 360 | 40031.71 | 8.83 | -0.869 | nucleus |
| MS.gene90044.t1 | *MsSPL20-4* | 73321209 | 73323721 | 309 | 34296.56 | 9.29 | -0.718 | nucleus |
| MS.gene044467.t1 | *MsSPL21-1* | 15915223 | 15917814 | 470 | 52457.17 | 6.16 | -0.569 | nucleus |
| MS.gene033046.t1 | *MsSPL21-2* | 16189961 | 16192557 | 470 | 52486.21 | 6.09 | -0.564 | nucleus |
| MS.gene063508.t1 | *MsSPL21-3* | 17919369 | 17921960 | 470 | 52486.21 | 6.09 | -0.564 | nucleus |
| MS.gene36036.t1 | *MsSPL22-1* | 52330951 | 52332049 | 180 | 20577.13 | 9.20 | -1.187 | nucleus |
| MS.gene032267.t1 | *MsSPL22-2* | 46790032 | 46791130 | 180 | 20577.13 | 9.20 | -1.187 | nucleus |
| MS.gene007908.t1 | *MsSPL22-3* | 47026334 | 47027433 | 180 | 20607.16 | 9.20 | -1.201 | nucleus |
| MS.gene34240.t1 | *MsSPL22-4* | 47265683 | 47266780 | 180 | 20557.12 | 9.19 | -1.190 | nucleus |
| MS.gene61571.t1 | *MsSPL23-1* | 83866879 | 83868787 | 334 | 36988.38 | 8.75 | -0.937 | nucleus |
| MS.gene000259.t1 | *MsSPL23-2* | 81917114 | 81919004 | 311 | 34492.63 | 8.55 | -0.970 | nucleus |
| MS.gene022540.t1 | *MsSPL23-3* | 80839494 | 80841343 | 311 | 34418.59 | 8.25 | -0.945 | nucleus |
| MS.gene62778.t1 | *MsSPL23-4* | 80871160 | 80873005 | 314 | 34850.08 | 8.54 | -0.952 | nucleus |
