# Supplementary material for: Identification of Alfalfa SPL gene family and expression analysis under biotic and abiotic stresses
Source: Sci Rep. 2023 Jan 3;13:84. doi: 10.1038/s41598-022-26911-7 (PMC9810616; doi:10.1038/s41598-022-26911-7)
Supplement: Supplementary file 3 — Supplementary Information 3. [file 41598_2022_26911_MOESM3_ESM.docx]

**Supplementary Table 2** Secondary structure and tertiary structure prediction of MsSPL protein

| Gene ID | *Gene* | Alpha helix | Extended strand | Random coil | Tertiary structure |
| --- | --- | --- | --- | --- | --- |
| MS.gene026366.t1 | *MsSPL1-1* | 257 (25.27%) | 190 (18.68%) | 570 (56.05%) | 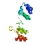 |
| MS.gene040800.t1 | *MsSPL1-2* | 241 (24.29%) | 186 (18.75%) | 565 (56.96%) | 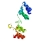 |
| MS.gene061710.t1 | *MsSPL1-3* | 242 (27.44%) | 130 (14.74%) | 510 (57.82%) | 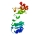 |
| MS.gene061104.t1 | *MsSPL1-4* | 259 (29.37%) | 126 (14.29%) | 497 (56.35%) | 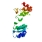 |
| MS.gene97631.t1 | *MsSPL2-1* | 39 (10.18%) | 73 (19.06%) | 271 (70.76%) | 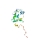 |
| MS.gene018772.t1 | *MsSPL2-2* | 29 (8.41%) | 71 (20.58%) | 245 (71.01%) | 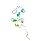 |
| MS.gene005475.t1 | *MsSPL3-1* | 290 (28.91%) | 178 (17.75%) | 535 (53.34%) | 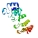 |
| MS.gene07353.t1 | *MsSPL3-2* | 285 (28.41%) | 178 (17.75%) | 540 (53.84%) | 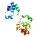 |
| MS.gene20896.t1 | *MsSPL3-3* | 285 (28.41%) | 178 (17.75%) | 540 (53.84%) | 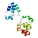 |
| MS.gene050360.t1 | *MsSPL3-4* | 282 (28.12%) | 178 (17.75%) | 543 (54.14%) | 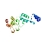 |
| MS.gene050361.t1 | *MsSPL3-5* | 58 (22.92%) | 50 (19.76%) | 145 (57.31%) | 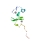 |
| MS.gene060699.t1 | *MsSPL4-1* | 30 (27.03%) | 28 (25.23%) | 53 (47.75%) | 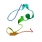 |
| MS.gene71881.t1 | *MsSPL4-2* | 30 (27.03%) | 28 (25.23%) | 53 (47.75%) | 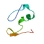 |
| MS.gene58356.t1 | *MsSPL4-3* | 30 (27.03%) | 28 (25.23%) | 53 (47.75%) | 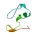 |
| MS.gene060698.t1 | *MsSPL5-1* | 21 (29.17%) | 18 (25.00%) | 33 (45.83%) | 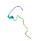 |
| MS.gene71880.t1 | *MsSPL5-2* | 21 (29.17%) | 18 (25.00%) | 33 (45.83%) | 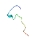 |
| MS.gene58357.t1 | *MsSPL5-3* | 21 (29.17%) | 18 (25.00%) | 33 (45.83%) | 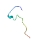 |
| MS.gene059775.t1 | *MsSPL5-4* | 21 (29.17%) | 17 (23.61%) | 34 (47.22%) | 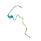 |
| MS.gene77091.t1 | *MsSPL6-1* | 67 (20.36%) | 54 (16.41%) | 208 (63.22%) | 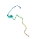 |
| MS.gene77090.t1 | *MsSPL6-2* | 67 (20.36%) | 54 (16.41%) | 208 (63.22%) | 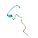 |
| MS.gene98509.t1 | *MsSPL7-1* | 226 (22.05%) | 171 (16.68%) | 628 (61.27%) | 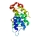 |
| MS.gene033954.t1 | *MsSPL7-2* | 236 (23.00%) | 157 (15.30%) | 633 (61.70%) | 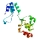 |
| MS.gene062030.t1 | *MsSPL7-3* | 230 (22.44%) | 168 (16.39%) | 627 (61.17%) | 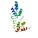 |
| MS.gene002799.t1 | *MsSPL8-1* | 197 (26.37%) | 149 (19.95%) | 401 (53.68%) | 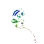 |
| MS.gene96180.t1 | *MsSPL8-2* | 194 (26.01%) | 152 (20.38%) | 400 (53.62%) | 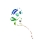 |
| MS.gene00967.t1 | *MsSPL8-3* | 195 (26.10%) | 149 (19.95%) | 403 (53.95%) | 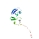 |
| MS.gene004483.t1 | *MsSPL8-4* | 195 (26.14%) | 149 (19.97%) | 402 (53.89%) | 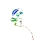 |
| MS.gene002482.t1 | *MsSPL9-1* | 28 (23.53%) | 28 (23.53%) | 63 (52.94%) | 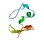 |
| MS.gene047335.t1 | *MsSPL9-2* | 37 (26.24%) | 27 (19.15%) | 77 (54.61%) | 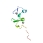 |
| MS.gene01060.t1 | *MsSPL9-3* | 16 (16.00%) | 28 (28.00%) | 56 (56.00%) | 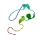 |
| MS.gene75620.t1 | *MsSPL10-1* | 56 (12.90%) | 74 (17.05%) | 304 (70.05%) | 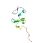 |
| MS.gene77585.t1 | *MsSPL10-2* | 56 (12.23%) | 88 (19.21%) | 314 (68.56%) | 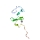 |
| MS.gene045290.t1 | *MsSPL10-3* | 56 (12.53%) | 81 (18.12%) | 310 (69.35%) | 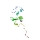 |
| MS.gene045288.t1 | *MsSPL10-4* | 56 (12.53%) | 82 (18.34%) | 309 (69.13%) | 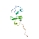 |
| MS.gene06231.t1 | *MsSPL11-1* | 29 (7.71%) | 92 (24.47%) | 255 (67.82%) | 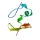 |
| MS.gene055507.t1 | *MsSPL11-2* | 45 (11.97%) | 88 (23.40%) | 243 (64.63%) | 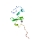 |
| MS.gene013155.t1 | *MsSPL11-3* | 45 (11.97%) | 88 (23.40%) | 243 (64.63%) | 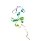 |
| MS.gene030453.t1 | *MsSPL12-1* | 36 (9.60%) | 90 (24.00%) | 249 (66.40%) | 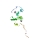 |
| MS.gene95236.t1 | *MsSPL12-2* | 36 (11.61%) | 69 (22.26%) | 205 (66.13%) | 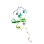 |
| MS.gene09220.t1 | *MsSPL13-1* | 79 (23.10%) | 63 (18.42%) | 200 (58.48%) | 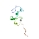 |
| MS.gene023426.t1 | *MsSPL13-2* | 84 ( 24.56%) | 61 ( 17.84%) | 197 ( 57.60%) | 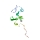 |
| MS.gene08539.t1 | *MsSPL13-3* | 84 ( 24.56%) | 61 ( 17.84%) | 197 ( 57.60%) | 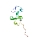 |
| MS.gene09027.t1 | *MsSPL13-4* | 187 (25.69%) | 119 (16.35%) | 422 (57.97%) | 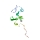 |
| MS.gene09219.t1 | *MsSPL14* | 78 (21.20%) | 64 (17.39%) | 226 (61.41%) | 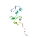 |
| MS.gene09218.t1 | *MsSPL15-1* | 48 (11.03%) | 96 (22.07%) | 291 (66.90%) | 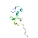 |
| MS.gene023428.t1 | *MsSPL15-2* | 19 (16.24%) | 26 ( 22.22%) | 72 (61.54%) | 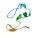 |
| MS.gene023425.t1 | *MsSPL15-3* | 97 (23.89%) | 64 (15.76%) | 245 (60.34%) | 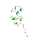 |
| MS.gene023424.t1 | *MsSPL15-4* | 52 (11.95%) | 98 (22.53%) | 285 (65.52%) | 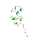 |
| MS.gene08540.t1 | *MsSPL15-5* | 78 (21.20%) | 64 (17.39%) | 226 (61.41%) | 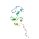 |
| MS.gene08541.t1 | *MsSPL15-6* | 52 (11.95%) | 96 (22.07%) | 287 ( 65.98%) | 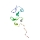 |
| MS.gene09028.t1 | *MsSPL15-7* | 52 (11.69%) | 96 (21.57%) | 297 (66.74%) | 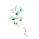 |
| MS.gene071085.t1 | *MsSPL16-1* | 115( 26.32%) | 80 ( 18.31%) | 242 ( 55.38%) | 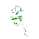 |
| MS.gene054760.t1 | *MsSPL16-2* | 102( 23.89%) | 80 ( 18.74%) | 245 ( 57.38%) | 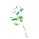 |
| MS.gene029630.t1 | *MsSPL16-3* | 102 (23.34%) | 81 ( 18.54%) | 254 ( 58.12%) | 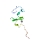 |
| MS.gene050425.t1 | *MsSPL17-1* | 269 (26.90%) | 172 (17.20%) | 559 (55.90%) | 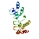 |
| MS.gene28650.t1 | *MsSPL17-2* | 271 (27.13%) | 168 (16.82%) | 560 (56.06%) | 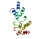 |
| MS.gene050856.t1 | *MsSPL17-3* | 274 (27.29%) | 170 (16.93%) | 560 (55.78%) | 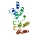 |
| MS.gene072658.t1 | *MsSPL17-4* | 263 (26.97%) | 167 (17.13%) | 545 (55.90%) | 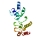 |
| MS.gene072657.t1 | *MsSPL17-5* | 274 (27.29%) | 170 (16.93%) | 560 (55.78%) | 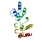 |
| MS.gene050857.t1 | *MsSPL17-6* | 273 (27.49%) | 167 (16.82%) | 553 (55.69%) | 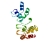 |
| MS.gene017969.t1 | *MsSPL18-1* | 48 (14.16%) | 82 (24.19%) | 209 (61.65%) | 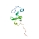 |
| MS.gene04608.t1 | *MsSPL18-2* | 48 (14.16%) | 79 (23.30%) | 212 (62.54%) | 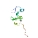 |
| MS.gene04607.t1 | *MsSPL18-3* | 48 (14.16%) | 79 (23.30%) | 212 (62.54%) | 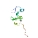 |
| MS.gene36181.t1 | *MsSPL19-1* | 69 (21.97%) | 83 (26.43%) | 162 (51.59%) | 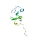 |
| MS.gene010796.t1 | *MsSPL19-2* | 65 (20.50%) | 93 (29.34%) | 159 (50.16%) | 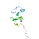 |
| MS.gene38329.t1 | *MsSPL19-3* | 21 (10.14%) | 77 (37.20%) | 109 (52.66%) | 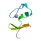 |
| MS.gene93246.t1 | *MsSPL19-4* | 64 (20.19%) | 93 (29.34%) | 160 (50.47%) | 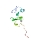 |
| MS.gene000446.t1 | *MsSPL20-1* | 79 (21.58%) | 68 (18.58%) | 219 (59.84%) | 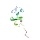 |
| MS.gene99828.t1 | *MsSPL20-2* | 77 (21.10%) | 64 (17.53%) | 224 (61.37%) | 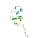 |
| MS.gene001948.t1 | *MsSPL20-3* | 77 (21.39%) | 63 (17.50%) | 220 (61.11%) | 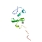 |
| MS.gene90044.t1 | *MsSPL20-4* | 72 (23.30%) | 51 (16.50%) | 186 (60.19%) | 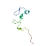 |
| MS.gene044467.t1 | *MsSPL21-1* | 58 (12.34%) | 103 (21.91%) | 309 (65.74%) | 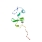 |
| MS.gene033046.t1 | *MsSPL21-2* | 58 (12.34%) | 104 (22.13%) | 308 (65.53%) | 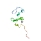 |
| MS.gene063508.t1 | *MsSPL21-3* | 58 (12.34%) | 104 (22.13%) | 308 (65.53%) | 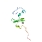 |
| MS.gene36036.t1 | *MsSPL22-1* | 56 (31.11%) | 26 ( 14.44%) | 98 (54.44%) | 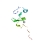 |
| MS.gene032267.t1 | *MsSPL22-2* | 56 (31.11%) | 26 ( 14.44%) | 98 (54.44%) | 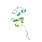 |
| MS.gene007908.t1 | *MsSPL22-3* | 55 (30.56%) | 26 (14.44%) | 99 (55.00%) | 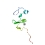 |
| MS.gene34240.t1 | *MsSPL22-4* | 60 (33.33%) | 29 (16.11%) | 91 (50.56%) | 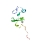 |
| MS.gene61571.t1 | *MsSPL23-1* | 66 (19.76%) | 47 (14.07%) | 221 (66.17%) | 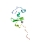 |
| MS.gene000259.t1 | *MsSPL23-2* | 76 (24.44%) | 36 (11.58%) | 199 (63.99%) | 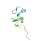 |
| MS.gene022540.t1 | *MsSPL23-3* | 66 (21.22%) | 44 (14.15%) | 201 (64.63%) | 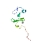 |
| MS.gene62778.t1 | *MsSPL23-4* | 66 (21.02%) | 43 (13.69%) | 205 (65.29%) | 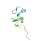 |
