# Supplementary material for: Identification of Alfalfa SPL gene family and expression analysis under biotic and abiotic stresses
Source: Sci Rep. 2023 Jan 3;13:84. doi: 10.1038/s41598-022-26911-7 (PMC9810616; doi:10.1038/s41598-022-26911-7)
Supplement: Supplementary file 6 — Supplementary Information 6. [file 41598_2022_26911_MOESM6_ESM.docx]

**Supplementary Table 5** Specific primers for *MsSPL* genes qRT-PCR analysis.

| Gene name | Forward primer sequence （5'→3'） | Reverse primer sequence（5'→3'） |
| --- | --- | --- |
| *GAPDH* | GGCTGCCATCAAGGAGGAAT | TCCAAGCTCAGCCTCATCAAG |
| *MsSPL3-3* | AGTCGCACAGACCGAATTGT | ACAACCAGGGCGTATGTAGC |
| *MsSPL1-4* | CACCCATCACCGGGAATTGA | AGCTCCGTGAACCGGAAAAT |
| *MsSPL4-1* | GGGGAAGCAAACATCAGTTGAG | ACTTGGCTCTTTTCTCCCACT |
| *MsSPL7-3* | AATCATTGTGGGCTCTGCCA | CATACAGCTGCAATGGCGAC |
| *MsSPL9-2* | TGCCAGCAATGTAGCAGGTT | ATTCAGACGTGCTTTTGCGG |
| *MsSPL5-4* | TGTGCGCAGGTTTCATGAGC | CAGCAGTGCTTTTACGACGC |
| *MsSPL10-4* | ACACTCCAATCGGACAACCG | GGAGCTGGCCTGATGTTGAT |
| *MsSPL15-2* | TGTCTCTGGTGAACCATTGCT | AGGTCAAGGCCACAACCTTC |
| *MsSPL15-5* | TTGCCAGCAATGTAGCAGGT | TATACGGCGCCTTGCATTGT |
| *MsSPL20-1* | TTGACTCGGGTTGTGCTCTC | GGAGTACGAGCACTCCTTCG |
| *MsSPL17-4* | CACTCCATATAGCAGCCGGG | CTGTCGCGAGCACTATTCCA |
| *MsSPL18-3* | CAGAGGTGGCAGCTTCTTGA | GTCCCACCTACCGAACCTTG |
| *MsSPL23-2* | CCCATAGACTTCGTGGGCTC | GAGGCGAGTTCGAAGAACCT |
| *MsSPL21-3* | CTGTGCCTGTCACCAATGGA | TGTAGCCAATGGCATGAGCA |
