# Supplementary material for: Identification of Alfalfa SPL gene family and expression analysis under biotic and abiotic stresses
Source: Sci Rep. 2023 Jan 3;13:84. doi: 10.1038/s41598-022-26911-7 (PMC9810616; doi:10.1038/s41598-022-26911-7)
Supplement: Supplementary file 7 — Supplementary Information 7. [file 41598_2022_26911_MOESM7_ESM.docx]

**Supplementary Table 6** Duplicated *MsSPL* genes and the divergence time of *MsSPL* genes

| Gene 1 | Gene 2 | Ka | Ks | Ka/Ks | MYA | Duplication Type |
| --- | --- | --- | --- | --- | --- | --- |
| *MsSPL1-1* | *MsSPL1-2* | 0.002 | 0.013 | 0.131 | 0.889 | Segmental |
| *MsSPL1-1* | *MsSPL1-3* | 0.008 | 0.021 | 0.377 | 1.374 | Segmental |
| *MsSPL1-1* | *MsSPL1-4* | 0.008 | 0.029 | 0.283 | 1.946 | Segmental |
| *MsSPL2-1* | *MsSPL2-2* | 0.022 | 0.069 | 0.319 | 4.584 | Segmental |
| *MsSPL3-1* | *MsSPL17-2* | 0.127 | 0.639 | 0.199 | 42.621 | Segmental |
| *MsSPL3-1* | *MsSPL17-1* | 0.128 | 0.636 | 0.201 | 42.407 | Segmental |
| *MsSPL1-2* | *MsSPL1-3* | 0.006 | 0.011 | 0.523 | 0.722 | Segmental |
| *MsSPL1-2* | *MsSPL1-4* | 0.006 | 0.019 | 0.320 | 1.285 | Segmental |
| *MsSPL3-2* | *MsSPL17-1* | 0.127 | 0.633 | 0.201 | 42.204 | Segmental |
| *MsSPL3-2* | *MsSPL17-2* | 0.127 | 0.636 | 0.199 | 42.418 | Segmental |
| *MsSPL3-4* | *MsSPL17-1* | 0.127 | 0.633 | 0.200 | 42.204 | Segmental |
| *MsSPL3-4* | *MsSPL17-2* | 0.126 | 0.636 | 0.198 | 42.418 | Segmental |
| *MsSPL1-3* | *MsSPL1-4* | 0.003 | 0.012 | 0.294 | 0.780 | Segmental |
| *MsSPL3-3* | *MsSPL17-1* | 0.127 | 0.633 | 0.201 | 42.204 | Segmental |
| *MsSPL3-3* | *MsSPL17-2* | 0.127 | 0.636 | 0.199 | 42.418 | Segmental |
| *MsSPL7-1* | *MsSPL7-3* | 0.010 | 0.051 | 0.191 | 3.421 | Segmental |
| *MsSPL7-1* | *MsSPL7-2* | 0.008 | 0.044 | 0.185 | 2.919 | Segmental |
| *MsSPL8-1* | *MsSPL8-3* | 0.002 | 0.018 | 0.098 | 1.187 | Segmental |
| *MsSPL8-1* | *MsSPL8-4* | 0.001 | 0.008 | 0.148 | 0.524 | Segmental |
| *MsSPL8-1* | *MsSPL8-2* | 0.005 | 0.036 | 0.129 | 2.401 | Segmental |
| *MsSPL9-1* | *MsSPL9-2* | 0.050 | 0.064 | 0.783 | 4.278 | Segmental |
| *MsSPL7-2* | *MsSPL7-3* | 0.009 | 0.057 | 0.163 | 3.832 | Segmental |
| *MsSPL8-2* | *MsSPL8-3* | 0.004 | 0.034 | 0.119 | 2.271 | Segmental |
| *MsSPL8-2* | *MsSPL8-4* | 0.003 | 0.028 | 0.125 | 1.860 | Segmental |
| *MsSPL8-3* | *MsSPL8-4* | 0.001 | 0.014 | 0.042 | 0.923 | Segmental |
| *MsSPL11-1* | *MsSPL11-3* | 0.020 | 0.033 | 0.606 | 2.175 | Segmental |
| *MsSPL11-1* | *MsSPL11-2* | 0.020 | 0.033 | 0.606 | 2.175 | Segmental |
| *MsSPL10-2* | *MsSPL10-3* | 0.002 | 0.003 | 0.582 | 0.222 | Segmental |
| *MsSPL13-1* | *MsSPL13-4* | 0.008 | 0.007 | 1.217 | 0.450 | Segmental |
| *MsSPL14* | *MsSPL13-4* | 0.003 | 0.005 | 0.551 | 0.303 | Segmental |
| *MsSPL15-1* | *MsSPL15-7* | 0.009 | 0.014 | 0.627 | 0.948 | Segmental |
| *MsSPL15-2* | *MsSPL15-5* | 0.139 | 0.372 | 0.373 | 24.824 | Tandem |
| *MsSPL15-2* | *MsSPL15-3* | 0.139 | 0.372 | 0.373 | 24.824 | Tandem |
| *MsSPL15-2* | *MsSPL14* | 0.139 | 0.374 | 0.371 | 24.960 | Segmental |
| *MsSPL13-2* | *MsSPL13-4* | 0.006 | 0.007 | 0.844 | 0.448 | Segmental |
| *MsSPL15-3* | *MsSPL13-4* | 0.005 | 0.004 | 1.097 | 0.274 | Segmental |
| *MsSPL15-4* | *MsSPL15-7* | 0.008 | 0.014 | 0.560 | 0.945 | Segmental |
| *MsSPL13-3* | *MsSPL13-4* | 0.006 | 0.007 | 0.844 | 0.448 | Segmental |
| *MsSPL15-5* | *MsSPL13-4* | 0.005 | 0.005 | 1.105 | 0.303 | Segmental |
| *MsSPL15-6* | *MsSPL15-7* | 0.009 | 0.014 | 0.630 | 0.945 | Segmental |
| *MsSPL16-1* | *MsSPL16-2* | 0.005 | 0.018 | 0.269 | 1.211 | Segmental |
| *MsSPL16-1* | *MsSPL16-3* | 0.012 | 0.063 | 0.186 | 4.231 | Segmental |
| *MsSPL16-2* | *MsSPL16-3* | 0.007 | 0.052 | 0.132 | 3.456 | Segmental |
| *MsSPL17-1* | *MsSPL17-2* | 0.002 | 0.013 | 0.160 | 0.899 | Segmental |
| *MsSPL19-1* | *MsSPL19-2* | 0.013 | 0.062 | 0.214 | 4.123 | Segmental |
| *MsSPL19-1* | *MsSPL19-4* | 0.015 | 0.083 | 0.176 | 5.538 | Segmental |
| *MsSPL20-1* | *MsSPL20-2* | 0.008 | 0.049 | 0.157 | 3.271 | Segmental |
| *MsSPL20-1* | *MsSPL20-3* | 0.005 | 0.054 | 0.099 | 3.570 | Segmental |
| *MsSPL20-1* | *MsSPL20-4* | 0.016 | 0.057 | 0.285 | 3.822 | Segmental |
| *MsSPL19-2* | *MsSPL19-4* | 0.007 | 0.038 | 0.179 | 2.557 | Segmental |
| *MsSPL20-2* | *MsSPL20-3* | 0.002 | 0.021 | 0.113 | 1.393 | Segmental |
| *MsSPL20-2* | *MsSPL20-4* | 0.020 | 0.054 | 0.366 | 3.629 | Segmental |
| *MsSPL17-4* | *MsSPL3-1* | 0.126 | 0.635 | 0.198 | 42.328 | Segmental |
| *MsSPL17-4* | *MsSPL3-2* | 0.125 | 0.634 | 0.197 | 42.277 | Segmental |
| *MsSPL17-4* | *MsSPL3-4* | 0.125 | 0.634 | 0.196 | 42.277 | Segmental |
| *MsSPL17-4* | *MsSPL3-3* | 0.125 | 0.634 | 0.197 | 42.277 | Segmental |
| *MsSPL17-4* | *MsSPL17-2* | 0.002 | 0.011 | 0.206 | 0.716 | Segmental |
| *MsSPL19-3* | *MsSPL19-4* | 0.026 | 0.044 | 0.582 | 2.942 | Segmental |
| *MsSPL19-3* | *MsSPL19-1* | 0.029 | 0.098 | 0.300 | 6.511 | Segmental |
| *MsSPL19-3* | *MsSPL19-2* | 0.027 | 0.056 | 0.480 | 3.717 | Segmental |
| *MsSPL20-3* | *MsSPL20-4* | 0.017 | 0.055 | 0.313 | 3.635 | Segmental |
| *MsSPL21-1* | *MsSPL21-2* | 0.002 | 0.003 | 0.570 | 0.214 | Segmental |
| *MsSPL21-1* | *MsSPL21-3* | 0.002 | 0.003 | 0.570 | 0.214 | Segmental |
| *MsSPL22-1* | *MsSPL22-3* | 0.002 | 0.037 | 0.064 | 2.462 | Segmental |
| *MsSPL22-1* | *MsSPL22-4* | 0.007 | 0.057 | 0.126 | 3.772 | Segmental |
| *MsSPL22-2* | *MsSPL22-3* | 0.002 | 0.037 | 0.064 | 2.462 | Segmental |
| *MsSPL22-2* | *MsSPL22-4* | 0.007 | 0.057 | 0.126 | 3.772 | Segmental |
| *MsSPL23-1* | *MsSPL23-2* | 0.003 | 0.017 | 0.153 | 1.146 | Segmental |
| *MsSPL23-1* | *MsSPL23-3* | 0.001 | 0.030 | 0.043 | 2.019 | Segmental |
| *MsSPL22-3* | *MsSPL22-4* | 0.009 | 0.057 | 0.168 | 3.772 | Segmental |
| *MsSPL23-2* | *MsSPL23-3* | 0.004 | 0.022 | 0.184 | 1.436 | Segmental |
